# Targeting Induced Local Lesions in the Wheat *DEMETER* and *DRE2* Genes, Responsible for Transcriptional Derepression of Wheat Gluten Proteins in the Developing Endosperm

**DOI:** 10.3389/fnut.2022.847635

**Published:** 2022-03-03

**Authors:** Nuan Wen, Claudia E. Osorio, Rhoda A. T. Brew-Appiah, Jaime H. Mejías, Tariq Alam, Samneet Kashyap, Steffen Reinbothe, Christiane Reinbothe, Charles P. Moehs, Diter von Wettstein, Sachin Rustgi

**Affiliations:** ^1^Department of Crop and Soil Sciences, Washington State University, Pullman, WA, United States; ^2^Instituto de Investigaciones Agropecuarias, INIA Carillanca, Temuco, Chile; ^3^Department of Plant and Environmental Sciences, School of Health Research, Clemson University Pee Dee Research and Education Centre, Florence, SC, United States; ^4^Laboratoire de Génétique Moléculaire des Plantes, Université Grenoble-Alpes, BP53F, Grenoble, France; ^5^Arcadia Biosciences, Davis, CA, United States

**Keywords:** wheat, celiac disease, gluten, epitope, reduced immunogenicity

## Abstract

Wheat is a major source of energy and nutrition worldwide, but it is also a primary cause of frequent diet-induced health issues, specifically celiac disease, for which the only effective therapy so far is strict dietary abstinence from gluten-containing grains. Wheat gluten proteins are grouped into two major categories: high-molecular-weight glutenin subunits (HMWgs), vital for mixing and baking properties, and gliadins plus low-molecular-weight glutenin subunits (LMWgs) that contain the overwhelming majority of celiac-causing epitopes. We put forth a hypothesis that eliminating gliadins and LMWgs while retaining HMWgs might allow the development of reduced-immunogenicity wheat genotypes relevant to most gluten-sensitive individuals. This hypothesis stems from the knowledge that the molecular structures and regulatory mechanisms of the genes encoding the two groups of gluten proteins are quite different, and blocking one group's transcription, without affecting the other's, is possible. The genes for gliadins and LMWgs have to be de-methylated by 5-methylcytosine DNA glycosylase/lyase (DEMETER) and an iron-sulfur (Fe-S) cluster biogenesis enzyme (DRE2) early during endosperm development to permit their transcription. In this study, a TILLING (Targeting Induced Local Lesions IN Genomes) approach was undertaken to identify mutations in the homoeologous *DEMETER* (*DME*) and *DRE2* genes in common and durum wheat. Lines with mutations in these genes were obtained that displayed reduced content of immunogenic gluten proteins while retaining essential baking properties. Although our data at first glance suggest new possibilities for treating celiac disease and are therefore of medical and agronomical interest, it also shows that inducing mutations in the *DME* and *DRE2* genes analyzed here affected pollen viability and germination. Hence there is a need to develop other approaches in the future to overcome this undesired effect.

## Introduction

Wheat is the primary staple or source of energy to more than one-third of the world population (1, 2). Carbohydrates (excluding non-starch polysaccharides), proteins, and dietary fibers like β-glucan and arabinoxylans, constitute 70–80%, 8–14%, and 3.5–4.0% of the dry matter in wheat grain, respectively (3, 4). Besides these macro-biomolecules, wheat also serves as a source of a wide range of health-beneficial phytochemicals and minerals, such as vitamins B, E, and A, anti-oxidants phytosterols, phenolic acids, alkylresorcinols, lignans, choline, and betaine, and microelements Ca, Mg, Fe, Zn, and Cu (5–7).

Other than the outlined benefits, wheat and derived products are also the elicitors of several frequent diet-induced health issues, specifically celiac disease (CD), wheat allergy, and non-celiac wheat sensitivity (NCWS), which collectively affect more than 7.5% of the US population (2, 8–10). Celiac disease alone affects about 1.4% of the world population and thus more than 110 million individuals, making it one of the most devastating gastrointestinal tract diseases worldwide (11).

The seed storage proteins of wheat, specifically prolamins (i.e., gliadins and glutenins), are known to possess both antigenic and allergenic properties. There are 630 prolamin sequences identified; 351 of these sequences correspond to gliadins and 229 to glutenins (12). An update to this database has been recently offered by Daly et al. (13), which brought the number of curated common wheat prolamin sequences to 699 (13).

Analysis of celiac causing epitopes indicated that gliadins and glutenins differ in their abilities to trigger the immunogenic reaction(s) in susceptible individuals. Also, gliadins showed great diversity and a high density of epitopes in sequences relative to LMWgs and HMWgs, which exhibited the lowest epitope density among other prolamins (13–15). This observation corresponds with the fact that LMWgs share ancestry with highly-immunogenic γ-gliadins, whereas the HMWgs form a phylogenetically distinct group (12, 13). Moreover, of all the known epitopes, only 25 epitopes belonged to the HMWgs, and only one of them exhibited immunogenic capacity causing a medium immune response (spot-forming unit values between 10 and 20), as determined using the Interferon-gamma (IFNγ)-ELISpot assay (15). Similarly, all epitopes that belonged to LMWgs exhibited weak immune responses. In contrast, the epitopes that belonged to gliadins (α-, γ-, and ω-gliadins) showed high immunoreactivity than epitopes identified in glutenins, particularly those that mapped to the A and D sub-genomes of common wheat (15).

The above information suggested the possibility to develop wheat genotypes with significantly reduced immunogenicity by targeted silencing of gliadin and LMWgs genes. Moreover, studies have shown that eliminating these proteins with imbalanced amino acid profiles (16) rather improves the flour lysine content due to the compensatory over-accumulation of albumins and globulins (17). Also, baking experiments performed on transgenic wheat lines deficient in different gliadin combinations (17–19) and with washed-out wheat flour residues mixed with known quantities of recombinant HMWgs (HMWDx5 and HMWDy10) demonstrated that eliminating gliadins and LMWgs do not detrimentally affect the baking properties of the wheat flour (20). Additionally, our earlier research demonstrated that the homoeologous wheat *DEMETER* (*DME*) and *Derepressed for Ribosomal protein S14 Expression* (*DRE2*) genes are responsible for the transcriptional activation of genes encoding gliadins and LMWgs in the developing wheat endosperm (21–23). Given this prior knowledge, we hypothesized that wheat genotypes with reduced immunogenicity and reasonable baking properties could be obtained by silencing the homoeologous wheat *DME* and *DRE2* genes using the TILLING (Targeting Induced Local Lesions IN Genomes) approach.

TILLING is a reverse genetic approach to identifying chemically induced mutations in known genomes (24–26). In the last two decades, this procedure has been extensively used in cereals and shown to be highly successful in isolating mutations in both common and durum wheat for several genes contributing to a variety of agronomically relevant traits (27–35), and has since remained one of the best methods of obtaining mutations in the desired genes.

For all of these reasons, this research aimed at isolating and characterizing TILLING mutations in the homoeologous wheat *DME* and *DRE2* genes using common and durum wheat populations. To pursue this goal, the following specific objectives were undertaken: (i) identification of mutations in the homoeologous wheat *DME* and *DRE2* genes from durum wheat “Kronos” and common wheat “Express” TILLING libraries; (ii) characterization of wheat TILLING mutants for their gluten content and composition; and (iii) pyramiding/stacking of mutants identified in individual *DME* or *DRE2* homoeologues to develop double mutants in durum wheat and triple mutants in common wheat with complete DME or DRE2 activity suppression.

## Materials and Methods

### Plant Material

Seeds of the selected *DRE2* TILLING mutants were obtained from the University of California, Davis, and seeds of the common wheat cultivar “Chinese Spring” nullisomic-tetrasomic lines lacking chromosome 5A, 5B, or 5D were obtained from the Wheat Genetics Resource Center (Kansas State University, Manhattan). The seeds of the diploid wheat progenitors [*Triticum urartu* (AA), *Aegilops speltoides* (BB), and *Aegilops tauschii* (DD)] and “Chinese Spring” [*Triticum aestivum* (AABBDD)] were obtained from the National Small Grains Collection (NSGC), Aberdeen, ID.

### EMS Mutagenesis, Plant Husbandry, and Sample Collection for DNA Extraction

Seeds of common wheat cultivar “Express” (WestBred) and durum wheat cultivar “Kronos” (Arizona Plant Breeders) were used to develop wheat TILLING populations. Wheat TILLING populations were generated by exposing Express and Kronos seeds to the mutagen ethyl methanesulfonate (EMS). Kronos seeds were treated with 0.75 or 1% EMS and Express seeds with 0.75, 1, or 1.2% EMS for 18 h [see (27) for details]. In a separate experiment, the Kronos seeds were treated with 0.7% (57 mM) or 0.75% (to 60 mM) EMS [cf. (36) for details]. Leaves from young M_2_ plants of each population were sampled for DNA extraction and screening for mutations in the homoeologous wheat *DME* and *DRE2* genes. Two to 20 M_3_ seeds (depending on the M_2_ plant zygosity) from selected genotypes were cultivated till maturity in 20 × 25 cm pots using Sunshine #1 potting mixture (SunGro Horticulture, Bellevue, USA) in a greenhouse maintained at a daytime temperature of 20–23°C and a night time temperature of 14–16°C with a photoperiod of 18 h. During this period, plants were fertilized weekly with nutrient water containing 200–250 ppm of water-soluble fertilizer [nitrogen (N), phosphorus (P_2_O_5_), and potassium (K_2_O)]. One-month-old M_3_ plant leaves were sampled for DNA extraction to determine the zygosity of mutant plants, and M_4_ seeds were harvested for protein profiling and determination of other end-use quality parameters.

### Screening of the TILLING Library

For large-scale TILLING library screening, DNA from two individual M_2_ plants was pooled. The DNA concentration for each individual within the pool was adjusted to about 2 ng/μL with a final concentration of 4 ng/μL for each pool. Twenty nanogram DNA (i.e., 5 μL of the pooled DNA samples) was arrayed on microtiter plates and subjected to gene-specific PCR. Amplification and TILLING were performed as described in Moehs et al. (34). The Kronos TILLING population developed at UC Davis was searched for mutations in the homoeologous *DRE2* genes *via* performing blast searches against the exon capture sequences derived from 1,535 EMS mutants (TILLING Genomic Ref. version 0.4 at https://dubcovskylab.ucdavis.edu/wheat_blast) using highly stringent search criteria [cf. (32)].

### Genomic DNA Extraction, PCR Amplification, and DNA Sequencing

Leaves of 3-week-old M_3_/M_4_ seedlings were collected in 96-well deep-well plates and dried for 72 h in closed tubes with silica gel. DNA was extracted from dried and finely ground samples using a modified cetyltrimethylammonium bromide (CTAB) method following Xin and Chen (37). DNA was treated with RNase and purified using the phenol: chloroform: isoamyl alcohol (25:24:1) precipitation. Then, the concentration of DNA samples was determined and adjusted to 50 ng/μL.

To determine the zygosity of individual M_3_/M_4_ plants, the target region from each line was amplified *via* PCR, and the amplicons were gel purified using the Geneclean kit following the manufacturer's instructions (MP Biomedicals, CA, USA). The eluted DNA was used as the template for the sequencing reaction using either forward or reverse primer in separate reactions. Sequencing reactions were carried out in 10 μL volume, and each reaction contained 100 ng DNA, 0.5 μM forward or the reverse primer, and BigDye mixture (Applied Biosystems, CA, USA). The following protocol was used for PCR: 96°C for 10 s, 50°C for 15 s, 60°C for 6 min with 24 iterations. After PCR, the product was column purified and resolved on the ABI 3730 DNA Analyzer. The DNA sequences were analyzed using the Sequencher 4.9 software (Gene Codes Corporation, MI, USA) to determine each line's genotype.

Genomic DNA was extracted from wheat cultivar “Chinese Spring,” the nullisomic-tetrasomic lines lacking chromosome 5A, 5B, or 5D in the Chinese Spring background and month-old plants of diploid wheat progenitors *T. urartu, Ae. speltoides*, and *Ae. tauschii*. After quantification, DNA was used to amplify *DME* genes from specific homoeologous chromosomes using sub-genome specific primers.

### RNA Isolation, cDNA Synthesis, and Real-Time PCR Assays

RNA was extracted from developing T_4_/T_5_ grains 17 days after anthesis (±3 days) when the immature grains were 50–75% filled. The spikes were harvested in liquid nitrogen and about 0.3 g of the developing grains was pulverized to fine powder and total RNA was extracted with TRIzol reagent (15596-018, Invitrogen) according to the manufacturer's recommendations. RNA was quantified using Bio-Rad SmartSpec 3000 (Bio-Rad Laboratories), and ~1 μg of RNA was converted to cDNA using a reverse transcription system with random oligos following the manufacturer's instructions (A3500, Promega).

The qRT-PCR analysis was performed using the LightCycler DNA Master SYBR Green I system (12158817001, Roche Diagnostics) on the LightCycler^®^ 480 Real-Time PCR System (05015278001, Roche Diagnostics) with the wheat *DME* primers (non-specific to homoeologous genes) 5′ TGTGCGTCTTTTGACACTCC 3′ and 5′ GCTCGTACAATGTCCGTTGA 3′ and *Actin* specific primers 5′ CACACTGGTGTTATGGTAGG 3′ and 5′ AGAAGGTGTGATGCCAAAT 3′ strictly following Wen et al. (21). *DEMETER* mRNA level was normalized to *Actin* using the DDC_T_ method. Transcript levels were expressed as a *DME* transcripts (normalized to *Actin*) ratio in control Kronos or Express plants to the EMS mutants.

### Gliadin and Glutenin Extraction From the Endosperm

Gluten proteins were extracted from the mature grains of the M_4_/M_5_ lines and wild type controls (Kronos and Express). In each case, three to four random grains were used for gluten extraction. For this purpose, seeds were dissected into the endosperm part and the germ part, the germ part was saved for plant propagation in the greenhouse, and the endosperm part was used for sequential gliadin and glutenin extraction.

The endosperm of the single seed was ground to flour using pestle and mortar. The amount of flour was determined by weighing, and 210 μL of 60% ethanol (v/v) was added to the flour samples weighing >20 mg and 100 μL of 60% ethanol to flour samples weighing <20 mg. In most cases, the endosperm-half of a single seed weighed around 20–80 mg. After ethanol addition, tubes were vortexed for 45 s to mix the flour with ethanol and incubated at room temperature with constant shaking at 200 rpm for 1 h in an orbital shaker. After incubation, samples were centrifuged at 8,200 rpm for 20 min, and the supernatant (carrying gliadins) was transferred to a new tube and stored at 4°C.

The pellet from the previous step was used for glutenin extraction. Two hundred and thirty microliter of freshly prepared glutenin extraction buffer [60% 1-propanol (v/v), 2 M urea (w/v), 0.05 M Tris-HCL (pH 7.5), 2% dithiothreitol (DTT) (w/v)] was added to the tube. Since DTT is unstable at room temperature, a fresh solution was made each time and added to the extraction buffer. The pellet was resuspended in the buffer by vortexing the tubes for 45–60 s or until a homogeneous mixture was achieved and incubated at 60°C with constant shaking at 200 rpm for 2.5 h in an orbital shaker. Subsequently, the mixture was centrifuged at 8,000 rpm for 20 min, then at 13,000 rpm for 2 min at room temperature. The higher centrifugation speed used in the second step allowed the pellet to stick to the tube wall and facilitated supernatant collection. The supernatant was collected (carrying glutenins) to a new tube and stored at 4°C until used.

### Polyacrylamide Gel Electrophoresis of Gliadins and Glutenins

The gliadins (the *DRE2* TILLING mutants and wild type Kronos) and glutenins extracted from mutant and wildtype lines (three biological replicates) were resolved on a 10% SDS-PAGE gel following Mejías et al. (38). Briefly, SDS-PAGE gels used in the present study consisted of two layers, stacking gel and separating gel. The separating gel was prepared by mixing 4 mL of acrylamide-bisacrylamide stock solution [30% acrylamide (w/v) and 0.78% bisacrylamide (w/v)] with 5 mL of distilled water, 3 mL of 3 M TRIS-HCL (pH 8.8), 120 μL of 10% SDS (w/v), 120 μL of 10% APS (w/v), and 6 μL of TEMED (Tetramethylethylenediamine). The stacking gel was prepared by mixing 1 mL of acrylamide-bisacrylamide stock solution with 4.25 mL of distilled water, 750 μL of 1M TRIS-HCL (pH 6.8), 60 μL of 10% SDS (w/v), 60 μL of 10% APS (w/v), and 4 μL of TEMED. After electrophoresis and staining, the gels were visualized on a lightbox and documented as mentioned in Mejías et al. (38).

Aluminum lactate polyacrylamide gels were prepared following Mejías et al. (38) with minor modification and used to profile the gliadin extracts from the *DME* TILLING mutants and wild type Kronos and Express. Gliadin samples were prepared for electrophoresis by adding 8 μL of 5 × loading buffer (containing 5 g sucrose and 2 mg of methyl violet in 10 mL of water) to 15 μL of gliadin extract before loading. Electrophoresis was performed at 500 volts (40 mA/gel) for 120 min using the SE 600 Ruby system (80-6479-57, GE Healthcare Life Sciences) in the lactate buffer, pH 3.1 at 15°C. After electrophoresis, the gels were equilibrated for 30 min in 10% trichloroacetic acid (TCA). A 0.5% solution Coomassie Brilliant Blue R-250 (BP101-25, Fisher Scientific) in ethanol was then added. The gels were stained with gentle shaking for 18 h at room temperature, and then each gel was destained in 450 mL of distilled water with 4 drops of Triton X-100 for 30 min. After staining, the gels were visualized on a lightbox and documented with a scanner.

### Reversed-Phase High-Performance Liquid Chromatography of Gliadins and Glutenins

In preparation for HPLC analysis, both gliadin and glutenin extracts from the *DME* TILLING mutants and wild type Kronos and Express were passed through 0.45 μm Durapore^®^ 13 mm membrane filters. The filtered samples were then injected onto a reversed-phase C8 analytical column (Zorbax 300SB-C8, Agilent Technologies) with 5 μm particle size and 30 nm microporous silica diameter (250 mm length, 4.6 mm I.D.), using a 1200 Series Quaternary LC-System liquid chromatograph (Agilent Technologies), with a diode array UV-V detector. The column temperature was maintained at 60**°**C, and a linear elution gradient was implemented using two mobile solvents. The polar solvent A consisted of a mixture of 0.1% trifluoroacetic acid (TFA) (v/v) and type-I ultrapure water (18 MΩ·cm specific resistance), and the non-polar solvent B contained 0.1% TFA (v/v) and acetonitrile (ACN). Absorbance was monitored at a detection wavelength of 210 nm. The injection volumes for both the gliadin and the glutenin fractions were 30 μL, and the flow rate was adjusted to 1.0 mL min^−1^. The elution gradient conditions were as described in Mejías et al. (38).

### Relative Quantification of Gliadin and Glutenin Fractions

HPLC was used to determine relative quantities of gliadins and glutenins in a sample. To obtain a standard curve using HPLC, increasing concentrations of bovine serum albumin (BSA; 0, 10, 25, 50, 75, 100, 150, and 300 mg/mL) were loaded onto the C8 reversed-phase analytical column (Zorbax 300SB-C8, Agilent Technologies) and resolved following Mejías et al. (38). Two peaks were observed, respectively, at 16.78 and 17.98 min retention times. Peaks were integrated, and the obtained peak areas in milli-absorbance units (mAU) were regressed against the BSA concentrations loaded onto the HPLC column to obtain a standard curve. The gliadin and glutenin extracts of the control and *DME* TILLING mutants were analyzed analogously as mentioned above for BSA, and the peak areas were calculated. The mAU values were plotted on the standard curve to determine relative protein concentrations, and the values obtained for each peak were summed to calculate a cumulative concentration for each of the glutenin and gliadin fractions from control and mutant lines.

### Densiometric Analysis

Gel images were captured on a lightbox using a Canon DS127721 DSLR camera. Gel images were all set to the same brightness and contrast setting using Adobe Photoshop ver. 20.0.06. Densitometric quantification of the protein bands was performed using ImageJ software (39) following (38). Briefly, each image was transformed to 8-bits. Subsequently, the image background was subtracted, and the optical density (OD) for each protein band was determined. The OD of each band shows up as a peak on the densitogram, where the area under each peak corresponds with the OD of the protein bands.

### Protein Content and Thousand-Kernel Weight

Seven seeds each of the wild type control and the *DME* TILLING mutant lines were used for studying the total grain protein content (GPC) using the LECO FP-228 Combustion N analyzer (FP228, LECO Corporation). The thousand-kernel weight was determined as an estimation of the grain yield.

### Quality Tests for the Control and the *DME* TILLING Mutants

Three procedures were applied to estimate the baking quality of the wildtype control and the *DME* TILLING mutants. (i) 500 mg of ground seeds from each genotype were used in the SDS sedimentation test as described by Carter et al. (40). (ii) 200 mg of ground seeds from each line were used in the sodium carbonate solvent retention capacity (SRC) test. The requisite weighed sample was added to a pre-weighed tube, and then 1 mL of a 5% (w/w) sodium carbonate solution was added. The mixture was vortexed and then allowed to gently and continuously agitate on a Labquake rotator for 20 min. The samples were then centrifuged at 1,000 × g for 15 min and the supernatant decanted. The tubes were inverted for 10 min and then swabbed with cotton to remove any extra solvent. The tubes were then weighed again, and the weight of the flour was determined by subtracting the tube's weight from that of the tube and flour. The weight of the dry flour and the solvated flour or gel's weight was then used to calculate the SRC for each sample. (iii) 200 mg of ground seeds from each genotype were used in the sucrose SRC test. This test resembles the sodium carbonate SRC test, except in this case, instead of adding 1 mL of a 5% (w/w) sodium carbonate solution, 1 mL of a 50% (w/w) sucrose solution was added to samples, and the retention of sucrose in flour was studied.

### Pyramiding of Mutations by Genetic Crosses

Mutations in the single homoeologous *DME* genes on the “A” and “B” sub-genome of bread and durum wheat were crossed in combinations to obtain *DME* double mutants. All crosses were made reciprocally and in duplicates. The F_1_ and later F_2_ seeds were obtained for 3 and 4 different mutant combinations, respectively, in Kronos and Express backgrounds. The mutations for stacking were selected based on the type (substitution, splice site variation, or premature stop codon), locations in the *DME* active site, and effect on gluten accumulation in the wheat endosperm. The F_2_ grains obtained from the aforementioned crosses were propagated in 48 well-flats and checked for zygosity of double mutations by PCR followed by DNA sequencing.

## Results

### Cloning of the Homoeologous Wheat *DEMETER* Genes, Their Chromosome Assignment, and Development of Homoeologue-Specific Primers for Use in TILLING

**Figure 1:**
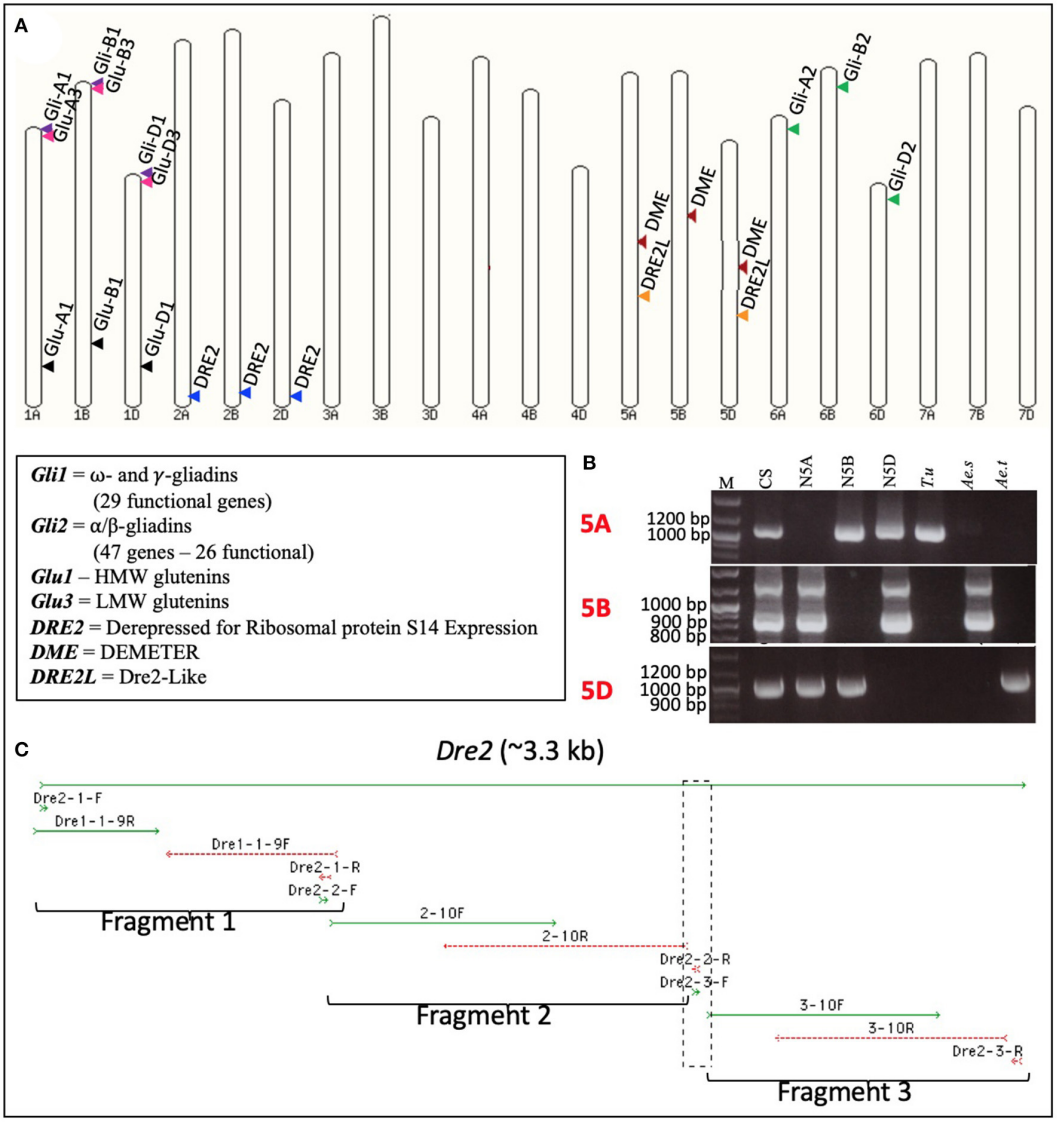
Subgenome and chromosome locations of wheat *DEMETER* (*DME*), *DRE2*, and *DRE2L* genes. Diagrammatic representation of the locations of the wheat *DEMETER* (*DME*), *DRE2, DRE2L*, and prolamin genes on wheat pseudochromosomes. **(A)** Gene locations were marked *via* arrowheads, and homoeologous genes were shown in similar colors. PCR amplification profiles obtained using the wheat *DME* homoeologues-specific primers (Supplementary Table S1) on the genomic DNA of wheat diploid progenitors - *Triticum urartu* (*T.u*), *Aegilops speltoides* (*Ae.s*), and *Aegilops tauschii* (*Ae.t*) - wheat nullisomic tetrasomic (NT) lines [group 5 chromosomes – null 5A (N5A), null 5B (N5B), and null 5D (N5D)] and the common wheat cultivar “Chinese Spring” (CS). **(B)** Notice the absence of bands in the NT lines and their presence in the corresponding subgenome. It confirms the presence of a gene in a certain wheat subgenome and a chromosome; *M* = molecular weight marker. Full-length sequence of the wheat *DRE2* homoeologues obtained using a stepwise cloning procedure **(C)**.

A pair of *DME*-specific primers (potentially amplifying the homoeologous wheat *DME* genes) covering nucleotide positions 13,263–13,618 on barley *DME* sequence FM164415.1 was designed and used to amplify fragment(s) from wheat genomic DNA. The PCR product was used to screen the common wheat (*Triticum aestivum* L.) cv. “Chinese Spring” BAC library, with ca. 1.3 million clones. Macroarray hybridizations led to the identification of three unique BAC clones, namely 1946D08, 2106P11, and 2159B03 (Supplementary Figure S1). Subsequently, the BAC clones were sequenced at >60-fold coverage by the 454-sequencing method. A total of 38.9 Mb of good quality sequence was obtained. Analysis of these BAC sequences revealed that each of them harbors a full-length *DME* sequence (accession numbers JF683316-JF683318). However, the three *DME* sequences differed from each other in length and composition. The observed differences in the length of *DME* homoeologues were mostly due to insertions and deletions (InDels) in the introns. Many point mutations [135 Homoeologous Sequence Variants (HSVs)] and small InDels observed among the *DME* homoeologous belonged to exons. These HSVs were used to design homoeologue-specific primers by tagging their 3′-ends at HSVs, and these primers were used to assign the *DME* homoeologues to specific sub-genomes (Supplementary Table S1). For this purpose, each homoeologue-specific primer pair was used on the genomic DNA of diploid wheat progenitors [*T. urartu* (AA), *Aegilops speltoides* (BB), and *Ae. tauschii* (DD)] with Chinese Spring [*T. aestivum* (AABBDD)] as control. These primers allowed unambiguous assignment of 2159B03 to chromosome 5A, 1946D08 to chromosome 5B, and 2106P11 to chromosome 5D (Figure 1). Sub-genome assignment of the *DME* homoeologues was further validated by the use of wheat group 5-specific nullisomic-tetrasomic lines (Figure 1).

### Cloning of the Homoeologous Wheat *DRE2* Genes

The DRE2 or Derepressed for Ribosomal protein S14 Expression gene facilitates the deposition of the iron-sulfur (Fe-S) cluster into the DME apoenzyme, which is vital for its interaction with genomic DNA and the DNA's subsequent demethylation. Given DRE2's role in DME activation, we decided to test its effect on prolamin accumulation. As a first step, we obtained the full-length DNA sequences of the wheat *DRE2* homoeologues following a comparative genomics approach. A two-step procedure was adopted as the *Arabidopsis DRE2* sequence (AT5G18400) did not yield a wheat sequence with apparent homology. Therefore, first, a putative *Arabidopsis DRE2* homolog was identified in rice and *Brachypodium*, then these sequences were used to identify homologous sequence(s) in common wheat. These searches allowed the identification of two homologs in rice (OS04G0674400 and OS04G0682050) and two homologs in *Brachypodium* (BRADI1G25576 and BRADI5G25970). When rice and *Brachypodium* sequences were blasted against the wheat reference genome sequence, the results suggested two homologs, one on group 2 chromosomes with a copy each on 2AL (TraesCS2A02G553500), 2BL (TraesCS2B02G585100), and 2DL (TraesCS2D02G555000), and another on group 5 chromosomes with a copy each on 5AL (TraesCS5A02G262300) and 5DL (TraesCS5D02G269900) (Figure 1). The copies on group 2 chromosomes closely resembled the rice and *Brachypodium DRE2* genes. Hence only this gene was considered further in this study. These *DRE2* homoeologues were amplified from durum wheat cv. “Kronos” in three overlapping fragments that covered the area from the transcription start site to the termination codon of the gene (Figure 1). The amplified fragments were cloned in the pGEM^®^-T Easy vector (Promega, Madison), and 20 clones per fragment were sequenced to have a fair representation of each homoeologue in the sequences. Obtained sequences were aligned with each other and the reference sequence. The sequence analysis revealed that we obtained the full-length sequence of the wheat *DRE2* homoeologues.

### Expression Pattern of the Wheat *DME, DRE2*, and the Immunogenic Prolamin Genes

**Figure 2:**
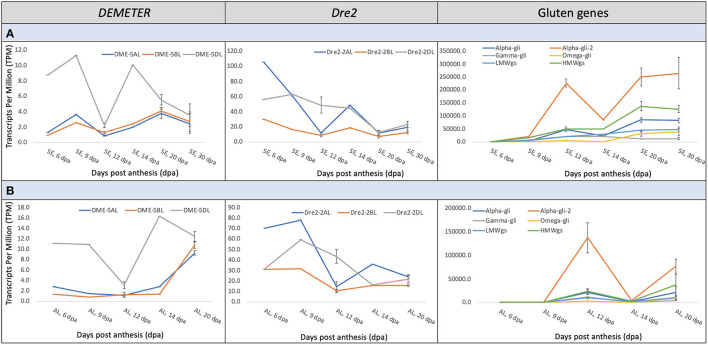
Meta-expression analysis of the wheat *DEMETER, DRE2*, and gluten genes at various grain developmental stages from 6–30 days post-anthesis (DPA) in starchy endosperm **(A)** and 6-20 DPA in the aleurone layer **(B)**. The data for expression analysis was derived from the Wheat Expression Browser. Notice the differences in the abundance of transcripts of different *DEMETER* and *DRE2* homoeologues and various grain developmental stages and their correspondence with the transcripts of wheat gluten genes.

Gene expression data across wheat grain development was accessed from the Wheat Expression Browser (http://www.wheat-expression.com) to understand the transcriptional interplay among the wheat *DME* and *DRE2* genes and genes encoding immunogenic gluten proteins (http://www.allergenonline.org/celiacproteinbrowse.shtml). This data is largely based on the study of Ramírez-González et al. (41). The *DME* and *DRE2* genes did not express in concert with each other. Similarly, the homoeologous copies of *DME* and *DRE2* exhibited asymmetrical expression patterns throughout the studied grain development period, i.e., 6–30 days post-anthesis (DPA) (Figure 2). The *DME* homoeologue on 5DL exhibited a dominant expression pattern throughout this period in the starchy endosperm and the aleurone layer (Figure 2). On the contrary, the *DRE2* homoeologues on 2AL and 2DL exhibited a developmental stage-specific asymmetrical expression pattern, where its 2AL-copy exhibited the highest expression at the 6 and 14 DPA in both starchy endosperm and aleurone and the 2DL-copy exhibited the highest expression at 12 DPA. In the starchy endosperm, the expression patterns of *DME* and *DRE2* seemed to somewhat follow a similar trend up to 20 DPA, whereas they exhibited opposite expression patterns in the aleurone. The expression patterns of these genes were compared with that of the expression patterns of immunogenic gluten proteins. An opposite expression trend was observed, which means the peaks of high gene expression for *DME* and *DRE2* opposed valleys of low gene expression for the immunogenic prolamins (Figure 2). For instance, the α-gliadins exhibited the highest expression during grain development with a peak at 12 DPA, whereas both *DME* and *DRE2* exhibited the highest expression, respectively, at 9 and 6 DPA. The second peak of α-gliadin expression was observed at 20 DPA and the third peak at 30 DPA, whereas for both *DME* and *DRE2*, their gene expression had started to decline at these developmental time points. The expression of other gliadins and glutenins started to rise at 30 DPA (Figure 2). In contrast, an opposite trend was observed for *DME* and *DRE2*. Among gluten proteins, the expression pattern of HMWgs genes somewhat followed the trend of the α-gliadins (Figure 2). The γ- and ω-gliadins showed an opposite expression pattern, and LMWgs showed a gradual increase in their expression between 6 and 30 DPA (Figure 2). In the aleurone layer, the expression patterns of *DRE2* and the immunogenic prolamin genes somewhat overlapped, but their expression was half that of their expression in the starchy endosperm (Figure 2).

**Figure 3:**
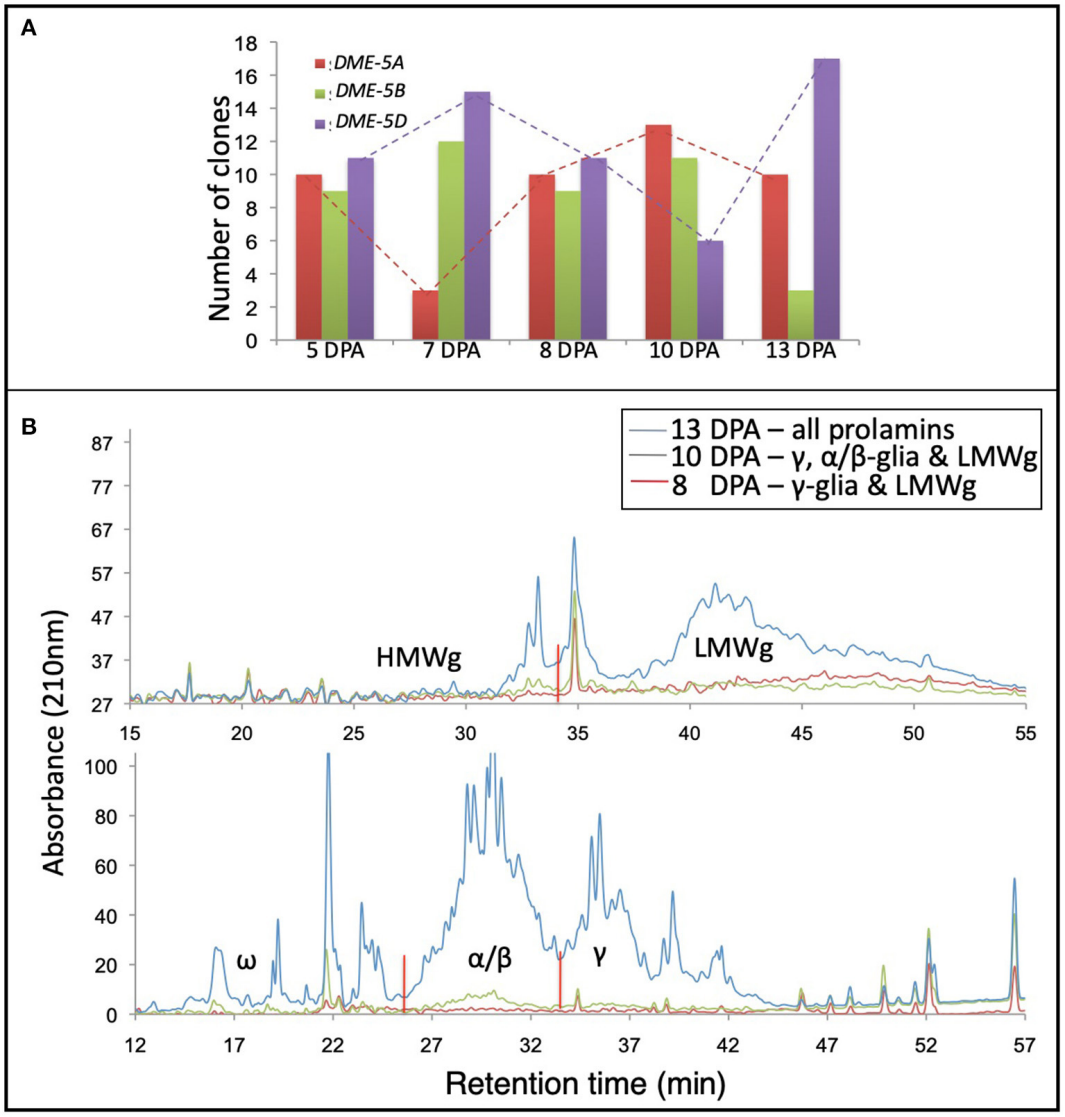
Coordinate expression of *DEMETER* and gluten proteins in the wheat grain. Picture showing the expression patterns of the wheat *DEMETER* homoeologues studied in developing endosperm at 5, 7, 8, 10, and 13 days post-anthesis (DPA) *via* PCR-cloning and DNA sequencing. **(A)** High-performance liquid chromatography of gliadins and glutenins from developing wheat grains at 8, 10, and 13 DPA. **(B)** Notice differential accumulation of different gliadins and glutenins in developing grains and the transcript abundance of different wheat *DEMETER* homoeologues.

Total RNA and gluten proteins were extracted from the developing wheat grains at 5, 7, 8, 10, and 13 DPA to validate the results of *in-silico* expression analysis. For the sake of precision about the anthesis date, wheat cv. “Express” spikes were emasculated and artificially pollinated with its pollen. Later, we collected the developing kernels in liquid nitrogen for expression analysis and protein profiling. For expression profiling of the homoeologous wheat *DME* genes, a set of primers (capable of amplifying all three *DME* homoeologues) were used to amplify products from cDNA, and the amplified product was cloned in a TA-cloning plasmid. Thirty colonies were sequenced *via* Sangers sequencing, and the expression level of different homoeologues was determined *via* sequence comparison with the genomic DNA sequences of the wheat *DME* homoeologues. At 5 and 8 DPA, the three homoeologous *DME* genes were expressed equally, but at the rest of the studied time points, the “A” and “D” subgenome *DME* homoeologues showed opposite expression patterns. In contrast to the “A” and “D” homoeologues, the “B” subgenome *DME* homoeologue maintained a constant expression level until 10 DPA (Figure 3).

Gliadins and glutenins were extracted from the developing grains, as described in the materials and methods, and resolved *via* HPLC. The protein quantity for seed samples collected at 5 and 7 DPA was too low to be resolved *via* HPLC; therefore, those samples were excluded from the analysis. At 8 DPA the γ-gliadins and LMWgs accumulated, and at 10 DPA γ- and α/β-gliadins and LMWgs accumulated, and by 13 DPA all prolamins accumulated (Figure 3).

Our earlier research established a general connection between the wheat *DME* expression and prolamin accumulation in the endosperm (21, 22). However, the relationship between the expression pattern of *DME* homoeologues and the accumulation of different prolamins during grain development was not studied. When the accumulation of prolamins was compared with the *DME* expression levels, it suggested that “B” and “D” subgenome *DME* homoeologues are largely responsible for initiating and maintaining transcription of genes encoding γ-gliadins at 8 DPA (Figure 3). By contrast, the “A” subgenome *DME* homoeologue, whose expression increases with time at 8 and 10 DPA, triggers the expression of genes encoding ω-gliadins and LMWgs (Figure 3). At the 13th DPA transcript of the “D” subgenome *DME* homoeologue reaches its maximum to induce expression of α/β-gliadin genes (Figure 3). This analysis suggests that the *DME* homoeologues have sub-functionalized to some extent during wheat evolution, but still retain some overlapping function.

### TILLING of *DME* Homoeologues in Tetraploid and Hexaploid Wheat

Tetraploid “Kronos” and hexaploid “Express” TILLING populations were screened for mutations in the wheat *DME* homoeologues. The average mutation density in the EMS mutagenized Kronos M_2_ population was 1 mutation per 40 kb DNA, and in the Express M_2_ population it was 1 mutation per 24 kb DNA (3). The mutations are primarily single nucleotide polymorphisms or small deletions.

**Figure 4:**
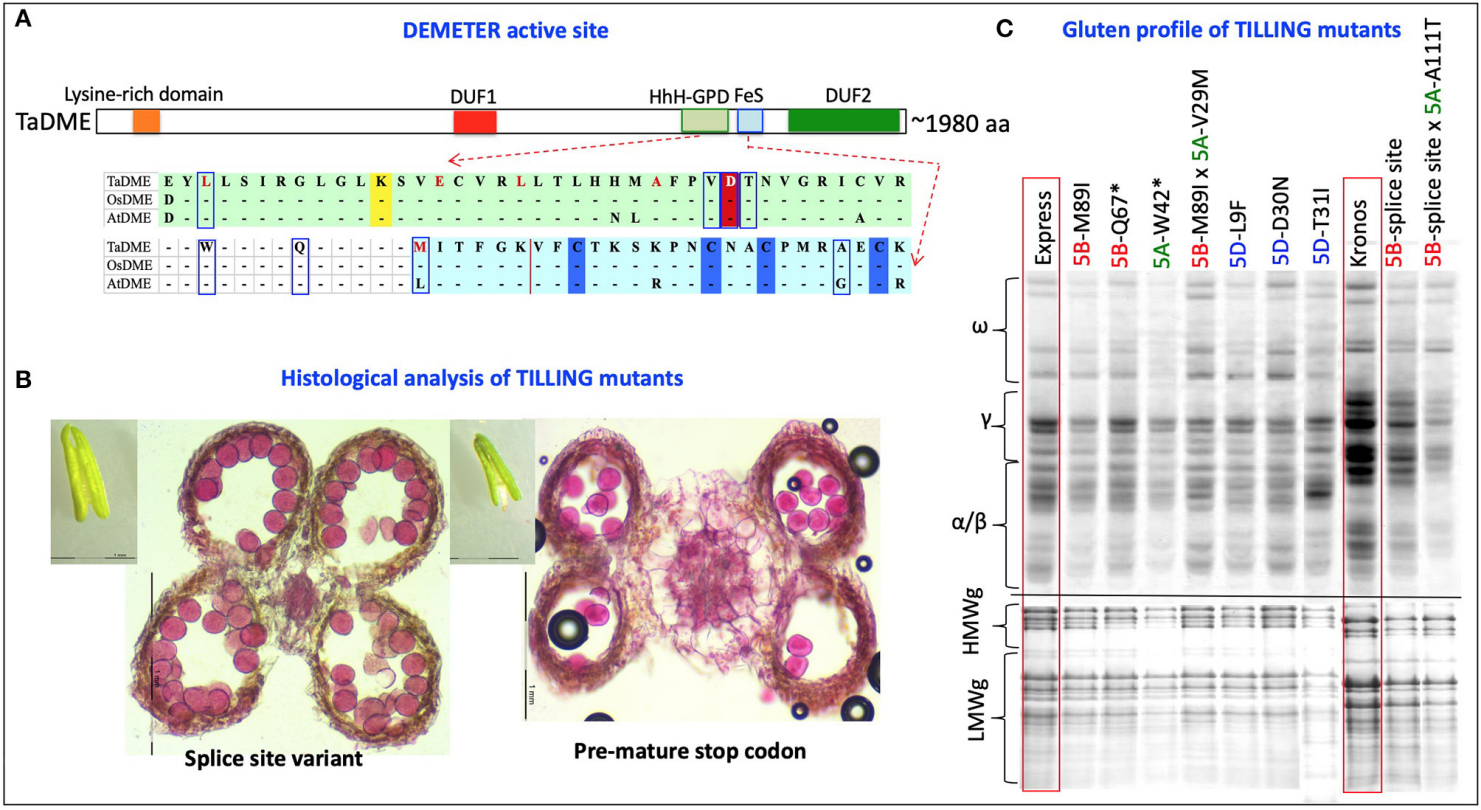
TILLING mutants of the Wheat *DEMETER* homoeologues. **(A)** Diagrammatic representation of the mutations identified in the active site of wheat *DEMETER* homoeologues. The glycosylase domain, including helix-hairpin-helix-Gly/Pro-rich loop followed by a conserved aspartate and iron-sulfur cluster, were highlighted by light green and light blue colors. Conserved lysine “K”, aspartic acid “D” and cysteine “C” residues are, respectively, highlighted by yellow, red, and blue colors. A dashed line indicates splice site variants. Mutations analyzed further for validation of mutant genotype and zygosity are boxed or underlined in splice site variants. Bold red font = mutations previously reported in *Arabidopsis* and shown to disrupt enzyme activity (42). **(B)** Differences were observed between the anthers and pollen grains of a splice site variant (left, 33,412) and a mutant with a premature stop codon (right, 36,505). Size of anthers was reduced to almost half in the mutant, “36505” carrying a stop codon in A sub-genome *DEMETER* homoeologue in “Kronos” background. To determine pollen viability, transversely sectioned anthers and isolated pollen grains were stained with a solution containing 10 mL of 95% alcohol, 1 mL of malachite green, 25 mL of glycerol, 5 mL of acid fuchsin, 0.5 mL of Orange G, and 4 mL of glacial acetic acid in 54.5 mL of distilled water. Pollens showing deep magenta color represent viable pollens. **(C)** Gliadin and glutenin profiles of selected TILLING mutants (M_3_) in “Express” and “Kronos” background showing reduced accumulation of gliadins and LMW glutenins in their grains. Two selected F_2_ lines from the genetic cross of “A” and “B” subgenome *DEMETER* mutants were also analyzed for their gluten profiles. *Premature stop codon.

Two runs, one each with a set of subgenome-specific primers, were executed on “Kronos” and “Express” M_2_ DNA bulks. The subgenome specific primers amplified 1,050 bp from the “A” subgenome of “Kronos” and “Express” and 1,044 and 855 bp from the “B” subgenome of “Kronos” and “Express,” respectively (Supplementary Table S1). The amplified region covered exon 5 to 9 of the genomic sequence containing the active site of the DME enzyme. In total, 42 mutations in “A” homoeologue and 35 mutations in “B” homoeologue of the durum wheat *DME* genes (*TdDME*) were detected. Among 42 mutations detected in the “A” homoeologue and 35 mutations detected in the “B” homoeologue, 4 and 5 mutations, respectively, coincided with the conserved motifs in the DNA glycosylase domain [consisting of a helix–hairpin–helix (HhH) motif, a glycine/proline-rich loop, and a conserved aspartic acid (GPD) residue]. A mutation resulting in a premature stop codon falling between the two conserved domains was also detected in the “A” homoeologue of *TdDME*. Similarly, 42 mutations in “A” homoeologue and 56 mutations in “B” homoeologue of the common wheat *DME* genes (*TaDME*) were detected (Table 1, Figure 4). Out of 42 mutations detected for the “A” homoeologue and 56 mutations detected for the “B” homoeologue, 6 and 5 mutations, respectively, coincided with the conserved domains, whereas four mutations, two each detected in “A” and “B” homoeologues resulted in either premature stop codons or splice site variants, that fell between the two conserved domains. Similarly, a “D” subgenome specific primer pair amplifying a 1,008 bp fragment from the common wheat genome was used to screen “Express” M_2_ DNA bulks. Three rounds of screening allowed the identification of 93 mutations in the “D” homoeologue of *TaDME*. Out of these 93 mutations, 11 coincided with the glycosylase domain (Table 1, Figure 4). One mutation resulted in a premature stop codon, and fell between the two conserved domains. Interestingly a point mutation at a conserved aspartic acid residue, repeatedly shown to disrupt DME enzyme activity in *Arabidopsis*, was also identified (43).

**Table 1:** Summary of mutations detected in the tetraploid and heaxploid wheat *via* TILLING.

**Cultivar**	**Sub-genome**	**Indivs. screened**	**Total (unique); frequency**	**Coding (unique); frequency**	**Non-synonymous/non-sense (unique)**	**Synonymous (unique)**	**Non-coding (unique); frequency**	**Active site**	**Between domains**
“Kronos”	A	3,456	42 (36); 1/86 kb	17 (15); 1/213 kb	9 (8)	8 (7)	25 (21); 1/145 kb	3 (S)	5
	B	3,840	35 (26); 1/115 kb	17 (16); 1/236 kb	10 (9)	7 (7)	18 (10); 1/223 kb	4 (S)	5
“Express”	A	3,456	42 (37); 1/86 kb	18 (16); 1/202 kb	9 (9)	9 (7)	24 (21); 1/151 kb	5 (S)	4 (S)
	B	3,456	56 (44); 1/53 kb	35 (26); 1/84 kb	25 (20)	10 (6)	21 (18); 1/141 kb	4 (S)	16
	D	13,824	93 (63); 1/150 kb	56 (34); 1/249 kb	28 (19)	28 (15)	37 (29); 1/377 kb	11 (S)	8

*S, splice site variant*.

A total of 344 M_3_ plants, including 130 plants, respectively, representing 3 and 7 (either in homozygous or heterozygous state) mutations in “Kronos” and “Express” backgrounds, underlying the conserved domains of “B” subgenome *DME* homoeologue, and 214 plants representing 5 and 9 mutants, respectively, in “Kronos” and “Express” backgrounds underlying the conserved domains of “A” subgenome *DME* homoeologue, were planted in the greenhouse. Similarly, a total of 44 M_3_ plants representing 9 (either in homozygous or heterozygous state) mutations in the “Express” background, underlying the conserved domains of “D” subgenome *DME* homoeologue, were propagated in the greenhouse. DNA was extracted from the leaves of 2 weeks old plants, used to amplify subgenome-specific *DME* products, and to determine the zygosity of plants by sequencing the PCR products (Supplementary Figure S2). The analysis allowed the identification of at least one homozygous M_3_ plant for each heterozygous M_2_ stock, except for the four cases where no homo/heterozygous mutant plant could be recovered (Supplementary Table S2).

### TILLING Mutants in the Durum Wheat *DRE2* Genes

A total of 36 mutations were identified in the 2AL *DRE2* homoeologue. Of these 36 mutations, ten mutations fell in introns, and 26 in exons−11 are synonymous variations, 12 are missense variations, two are amber mutations, and one is a splice site variant. Similarly, a total of 15 mutations were detected in the 2BL copy of the *DRE2* gene. Of these mutations, six fell in introns, five in exons, and four in the 3′-untranslated region. Two of these five mutations are synonymous variations, two are missense variations, and one is a splice site variant. Seeds of 13 *DRE2*-2AL mutants, including two amber mutants and 11 missense mutants, and three *DRE2*-2BL mutants with two missense mutants and a splice site variant were procured from the Dubcovsky Lab at UC Davis. Four to eight seeds of each line were propagated in the greenhouse, and the genotype of each mutant line was confirmed by DNA sequencing. At least one homozygous plant in each mutant was identified. All mutant lines were allowed to self-pollinate, and seeds from homozygous lines were used to determine the gluten profile.

### Transcriptional and Translational Suppression Observed for the Wheat *DME* and *DRE2* Genes and Their Effect on Gluten Protein Accumulation

Immature seeds (17 ± 3 DPA) from the homozygous M_3_ plants were used for RNA extraction, followed by qRT-PCR analysis. In reference to the wheat *Actin* gene, five *DME* mutants, namely 36505 (W42^*^), 42773 (V29M), and 33412 (splice site variant) in the “Kronos” background and 50324 (M89I) and 21599 (G93R) in the “Express” background exhibited transcriptional suppression (Supplementary Figure S3). On the other hand, none of the *DRE2* mutant lines exhibited any difference in their *DRE2* transcript levels.

Total grain protein from the 16 selected mutant lines, all carrying mutations in the active site region of *TaDME* and *TdDME*, were extracted from mature seeds and profiled using SDS-PAGE followed by densitometry. All genotypes showed reduced accumulation of one or more immunogenic gluten proteins. In particular, lines carrying premature stop codons (“Express” mutant “20396” in the “B”-subgenome and “12680” in the “A”-subgenome), splice site variant (“Kronos” mutant “33412” in the “B”-subgenome), or a non-synonymous substitution (“Express” mutant “50322” with M89I substitution in the “B”-subgenome, “13105” with L9F substitution, and “50449-6” with D30N substitution in the “D”-subgenome) showed significant reductions in gliadin and glutenin content (Figure 4, Supplementary Table S3). Gliadins and glutenins extracted from these mutants were also resolved using RP-HPLC. The area under peaks representative of different protein families on the chromatogram was converted to protein quantity in mg mL^−1^ compared to a standard curve developed using bovine serum albumin (BSA) resolved under similar conditions on RP-HPLC. The analysis suggested that in comparison to the wildtype “Express,” the single mutant “12680” (stop gained in “A” subgenome *DME* copy) accumulated significantly reduced amounts of HMWgs, LMWgs, and α/β-gliadins (about 81, 79, and 81% reductions, respectively). A similar great reduction in ω-gliadin content was observed in “Express” mutant “50322” (an M89I substitution in the “B”-subgenome *DME* copy) and γ-gliadins in “Express” mutant “50449” (a D30N substitution in the “D”-subgenome *DME* copy) (Table 2). Likewise, compared to wildtype “Kronos” we observed significant reductions in the content of HMWgs, LMWgs, ω-gliadins, and α/β-gliadins (68, 66, 60, and 44%, respectively) in mutant “33412” (a splice site variant in the “B”-subgenome *DME* copy) (Table 2).

**Table 2:** List of selected mutants in the wheat *DEMETER* genes showing reductions (in percent) in the content of various gluten proteins.

**Mutant ID**		**Glutenin subunit**		**Gliadin**		
		**HMW**	**LMW**	**ω**	**α/β**	**γ**
“Express”	Wildtype	0	0	0	0	0
N1	50322-80	61	61	71	64	70
N4	20396-66	60	58	55	56	51
N11	12680-67	81	79	51	81	73
N27	13105-1	71	69	62	65	71
N35	50449-6	38	65	32	52	75
“Kronos”	Wildtype	0	0	0	0	0
N46	33412-62	68	66	60	44	29
N22	12680-64	57	43	51	25	14
**Double mutants (derived** ***via*** **genetic crossing)**						
N25	50,322 × 20548-2	47	66	37	57	71
N54	33,412 × 29375-3	59	55	64	56	44
N56	33,412 × 29375-5	61	78	37	72	70

*The proteins were resolved via reversed-phase high-performance liquid chromatography (RP-HPLC), and their content was determined compared to the bovine serum albumin (BSA)*.

**Figure 5:**
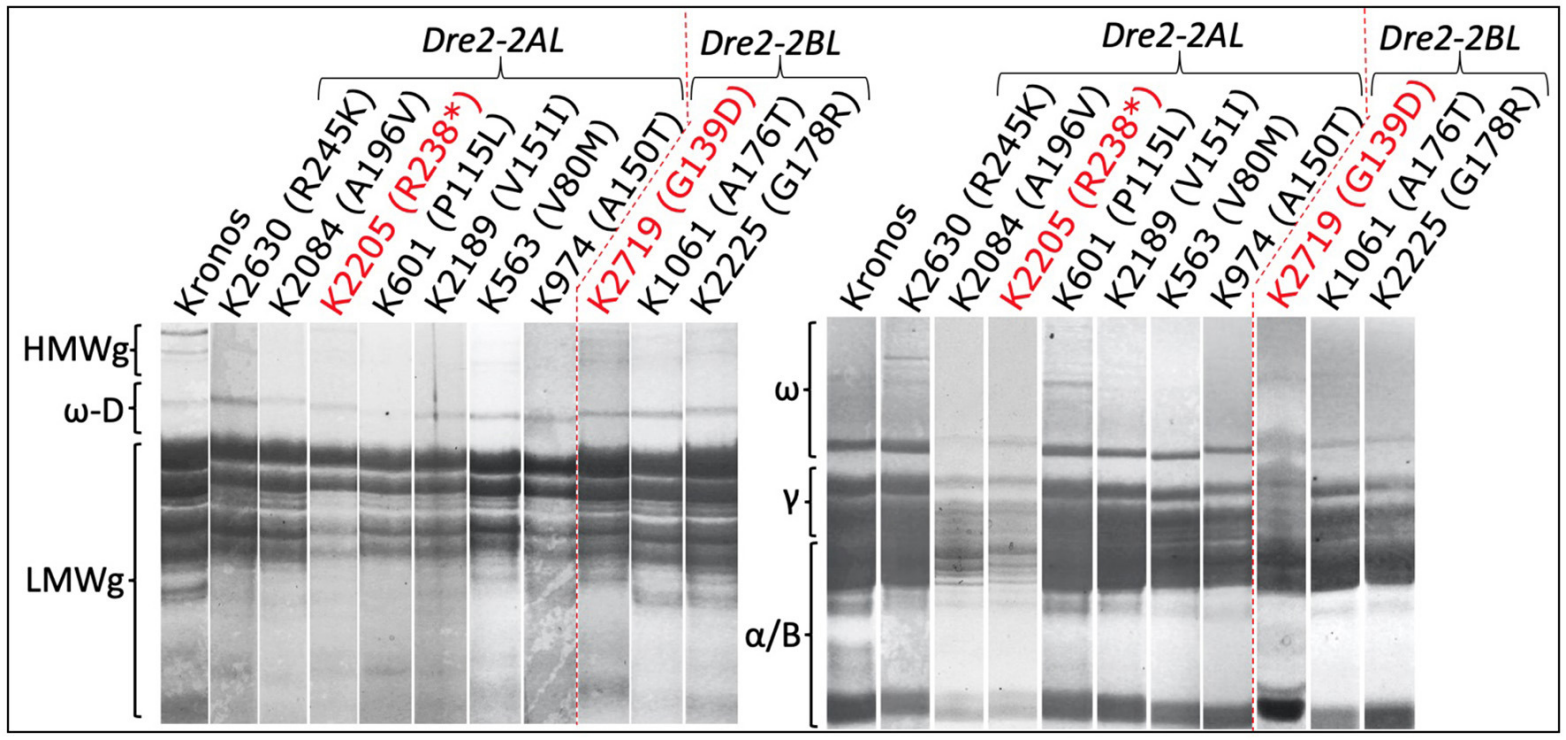
Sodium dodecyl sulfate-polyacrylamide gel electrophoresis of gliadin and glutenin proteins from the *DRE2* TILLING mutants. Notice the reduced or missing bands of different gliadins (right) and glutenins (left) in various *DRE2* mutants.

Three seeds (biological replicates) from 16 mutant lines (homozygous for mutations in the *DRE2* gene) were used to extract gliadins and glutenins, and the proteins were profiled using SDS-PAGE followed by densitometry. Protein profiles of mutants were compared with wild-type “Kronos” to study the effects of mutations in the individual homoeologous *DRE2* genes on chromosome arm 2AL and 2BL. Two types of differences were observed: qualitative differences and quantitative differences, where qualitative differences are the presence or absence of specific protein bands, and quantitative differences are under-accumulation or over-accumulation of the specific protein(s) (visible as reduced or high-intensity bands). Compared to “Kronos”, nine of 16 mutants showed quantitative differences, and seven mutants showed qualitative differences. Most if not all *DRE2* mutants exhibited reduced accumulation of ω- (27% reduction to complete lack) and γ-gliadins (40–93% reduction) and over-accumulation (range from 1.1 to 2.7 fold) of α/β-gliadins (except a non-synonymous T121I substitution in the *DRE2* 2AL homoeologue). Further, some mutants exhibited over-accumulation of HMWgs (range from 1.3 to 5.5 fold) and LMWgs (range from 1.1 to 2.2 fold), whereas some showed reduced HMWgs (14–78%) and LMWgs (6–44%) accumulation (Table 3, Figure 5).

**Table 3:** List of selected mutants in the wheat *DRE2* genes showing reductions (in percent) or over accumulations in the content of various gluten proteins.

**Mutant ID**	**Glutenin subunits**		**Gliadins**				**TKW (g)**
	**HMW**	**LMW**	**ω - Gliadins**	**ω-D Gliadins**	**γ - Gliadins**	**α/β - gliadins**	
**Dre2-2AL**							
SNV Kronos2205:G9670A	1.3 times	13	67	39	78	2.2 times	46.7
SNV Kronos604:G10513A	63	44	2.9 times	Missing	65	1.2 times	30.0
SNV Kronos2630:C9648T	64	34	2 times	1.9 times	27	1.2 times	50.0
SNV Kronos2084:G9912A	78	33	83	39	71	2.7 times	50.0
SNV Kronos601:G10126A	58	39	78	46	75	2.2 times	40.0
SNV Kronos2189:C10139T	36	24	91	59	83	1.9 times	30.0
SNV Kronos974:C10142T	73	6	Missing	Missing	90	2.3 times	70.0
SNV Kronos2312:C10190T	1.5 times	1.2 times	Missing	77	86	1.6 times	46.7
SNV Kronos382:G10304A	0	1.1 times	27	30	40	14	30.0
SNV Kronos3076:C10497T	14	1.1 times	Missing	68	84	1.3 times	30.0
SNV Kronos563:C10510T	5.5 times	1.4 times	67	34	53	1.6 times	43.3
SNV Kronos1214:G10600A	1.9 times	1.1 times	Missing	74	84	1.4 times	36.7
SNV Kronos799:C12741T	1.5 times	1.2 times	Missing	67	80	1.9 times	43.3
**Dre2-2BL**							
SNV Kronos2719:G3923A	1.8 times	5	2.1 times	1.2 times	3	20	33.3
SNV Kronos1061:G4033A	1.5 time	2.2 times	Missing	87	88	1.6 times	36.7
SNV Kronos2225:G4039A	3.8 times	1.8 times	Missing	94	93	1.1 times	33.3
“Kronos”	0	0	0	0	0	0	

*Missing, missing protein band(s)*.

*Fold increase in the gluten protein content is shown in times*.

*TKW, thousand-kernel weight. It is extrapolated from the weight of 100 kernels*.

*The proteins were resolved via sodium dodecyl sulfate polyacrylamide gel electrophoresis (SDS-PAGE), and their content was determined compared to the bovine serum albumin (BSA)*.

### Assessment of the End-Use Performance of the Reduced Gluten Wheat *DME* Mutants

We performed micro SRC and SDS sedimentation tests and determined the total grain protein content (GPC)—using the LECO FP-228 Combustion N analyzer (FP228, LECO Corporation)—of reduced-gluten *DME* mutants to predict their end-use performance. Kernel weight was determined for each line (from the seven randomly selected grains, also used later to determine the GPC); it ranged from 0.17 g in “Express” mutant “12680” to 0.31 g in “Express” mutant “20396”, which is significantly less or more than 0.29g in “Express”. The protein content expressed in percentage ranged from 13.17% in “Express” mutant “50449” to 26.45% in “Kronos” mutant “33412”, which is, respectively, less and more than “Express” (17.72%) and “Kronos” (22.68%). The components for diagnostic SRC solvents (water, dilute aqueous lactic acid, dilute aqueous sodium carbonate, and concentrated aqueous sucrose solution) were calculated using standard formula and assuming 14% water content of the flour. Values obtained using 50% sucrose ranged between 136% in “Express” mutant “50449” (similar to “Express”) to 178% in “Express” mutant “12680” and 143% in “Kronos” mutant “33412” (significantly <168% in “Kronos”), indicating higher water retention capacity in the finished bread. The results of the sodium carbonate test of the lines ranged from 129% in “Express” mutant “20396” to 196% in “Express” mutant “12680” (139% in “Express”) (Supplementary Table S4). The value of 146% for “Kronos” mutant “33412” is at par with “Kronos” (149%). It is an indicator of damaged starch, which leads to bread staling and reduced shelf life. Values for the lactic acid SRC ranged from 149% for “Express” mutant “50449” to 231% for “Express” mutant “12680,” which is significantly lower and higher than “Express” (180%). Similarly, for water SRC lowest (131%) and highest (214%) values were observed for “Express” mutants “50449” and “12680”, respectively. The gluten performance index (GPI) was calculated using the values of micro SRC. It is an indicator of the overall performance of flour glutenin in the presence of other flour polymers. The GPI ranges from 0.53 (“Express” mutant “50449”) to 0.76 (“Express” mutant “51464”) in the analyzed samples, lower than what is desired for bakers' flour (personal communication, Craig F. Morris; deceased October 25, 2021). Only two lines obtained values acceptable for bakers' flour, lines “20396” and “51464” with 0.71 and 0.76, respectively (Supplementary Table S4).

### Pyramiding of *DME* Single Mutants to Develop Double Mutants

The single mutants identified in the “A” and “B” subgenome *DME* homoeologues of common and durum wheat were crossed in combinations to obtain *DME* double mutants to stack their gluten phenotypes. Crosses were made reciprocally to account for possible maternal influence, which is a prerequisite, specifically when dealing with epigenetic regulators (Supplementary Figure S4). The mutants for crossing were selected based on the mutation type (substitution, splice site variation, or premature stop codon), their respective *DME* active site locations, and their gluten profiles. F_2_ seeds were obtained for three combinations in the “Kronos” background and four combinations in the “Express” background (Supplementary Figure S4). The F_2_ grains were planted (in 48-well flats) in the greenhouse and checked for zygosity at two *DME* loci *via* DNA sequencing. Sequence analysis allowed the identification of 20 double mutations in the “Kronos” background and 14 double mutants in the “Express” background (Supplementary Table S5). F_3_ grains were obtained from the double mutants.

All double mutants were identified as crosses between mutants carrying an amino acid substitution or a splice site variation in single *DME* homoeologues. However, even after screening a few hundred F_2_ plants, no double mutants were identified from the crosses between mutants carrying a premature stop codon in one homoeologue and an amino acid substitution in another or premature stop codons in both homoeologues in both “Express” and “Kronos” backgrounds. However, a double mutant with a splice site variant in one homoeologue and an amino acid substitution in another homoeologue was identified in the “Kronos” background.

Protein profiles of the mature F_3_ seeds, specifically a double mutant derived from a cross between “50322” (M89I, *DME*-5B) × “20548” (V29M; *DME*-5A) in the “Express” background, exhibited reduced accumulation of gliadins and LMWgs (greater reductions in ω- and α/β-gliadins and LMWgs, and less reductions in γ-gliadins) (Table 2). However, these reductions in gliadins and glutenins in the double mutant were relatively less apparent than in the single mutants with premature stop codons (Figure 4). The HPLC analysis of gluten proteins endorsed these observations. For instance, relative to “Express” (wildtype), a 50.8% reduction in ω-gliadins, a 43% reduction in LMWgs, and a 25.2% reduction in α/β-gliadins was observed (Table 2). On the other hand, the double mutant derived from a cross between “33412” (splice site, *DME*-5B) × “29375” (A111T, *DME*-5A) in the “Kronos” background exhibited a gluten profile reduced in gliadin and glutenin content more than any other single mutant (Figure 4). These results were supported by the results of HPLC analysis where >70% content reductions in α/β- and γ-gliadins and LMWgs were observed (Table 2). Preliminary analysis of the end-use performance of the double mutants “50322” × “20548” in the “Express” background and “33412” × “29375” in the “Kronos” background are at par with the respective wild type controls expect for the SDS sedimentation test values, which were twice as much (42 mm) as that of the control (24 mm) for the “50322” × “20548” double mutant and half that of the control (33 mm) for the “33412” × “29375” mutant (16 mm) (Supplementary Table S4).

The results of initial crosses made between the mutations in the “A” and “B” subgenome *DME* homoeologues showed distorted segregation in most of the combinations except for the cases where crosses were made between genotypes carrying amino acid substitutions (with mild effects on the gluten profiles). To determine the cause of the observed segregation distortion, we studied anther morphology, anatomy, number of viable pollen grains, and pollen germination (in pollen germination media supplemented with azadecalin) (44). Interestingly, in contrast to the barley high-lysine mutants, *DME* mutants carrying premature stop codons or splice site variants, especially in the tetraploid wheat background, showed reductions in anther size, locule size, number of fertile pollen grains, and pollen germination rate (Figure 4). The reduced pollen germination adversely affects the competitiveness of the mutant pollen grains relative to the wild-type pollen grains causing severe segregation distortion. Indeed, analysis of hundreds of F_2_ plants from crosses between “A” and “B” sub-genome *DME* mutants with premature stop codons yielded either single mutants or wild type alleles at both homoeologues. In addition, a reduction in seedling vigor of the *DME* mutants with stop codons was also observed.

Crosses between mutants belonging to other homoeologous groups were attempted; for instance, crosses were made between “A” and “D” and “B” and “D” subgenome *DME* homoeologues. When mutants carrying splice site variants and/or premature stop codons in different *DME* homoeologues were crossed in combinations, a limited number of grains were obtained. Unfortunately, none of the tested genotypes carried double mutations either in the homozygous or the heterozygous state. Similarly, the *DRE2* mutants (except four mutants) in either the 2AL copy or the 2BL copy due to their effect on pollen viability and anther size were maintained in the heterozygous state, making it challenging to obtain homozygous lines to make genetic crosses to stack their gluten phenotypes.

## Discussion

Wheat is an all-round and affordable source of nutrition (including micronutrients) for the global population. Fiber in wheat promote gut health as these are fermented by the gut microbiota into health-promoting phenolics and antioxidants (4, 45). The carbohydrates other than fibers are a great energy source, and the proteins, except for a deficiency in lysine and threonine, contain well-balanced amino acids (46). However, wheat seed proteins, specifically gluten proteins, exhibit incomplete digestibility due to repetitive motifs consisting primarily of proline and glutamine residues. In susceptible individuals, many indigestible peptides were identified to trigger immune responses, such as celiac disease, wheat allergy, and non-celiac wheat sensitivity (2). No therapy other than a restrictive diet is available, which is not a straightforward solution due to the ubiquity of gluten proteins in processed foods, medicines, and cosmetics, unintended contaminations, and fiscal/social reasons (47). Hence a broadly acceptable solution to this problem is sorely needed.

Gluten proteins constitute 75–80% of the wheat seed proteins on a dry matter basis. Based on two-dimensional (2D) electrophoresis, the numbers of gluten proteins present in wheat cultivars are estimated to be between 50 and 100 (48). Whereas based on mass spectrometry, Bromilow et al. (12) determined the numbers of gluten proteins in wheat to be 63. Therefore, the exact numbers of gluten proteins in a mature wheat grain indeed remains elusive (48). Interestingly, all of the different wheat gluten proteins are encoded by nine loci distributed on the group 1 and group 6 chromosomes of the three (“A,” “B,” and “D”) subgenomes in common wheat (Figure 1) and the two subgenomes (“A” and “B”) in durum wheat (4, 48). Each gluten locus comprises multiple genes (15). The simplest gluten locus is the *Glu-1* locus that maps to the long arms of the group-1 chromosomes (Figure 1), where each locus consists of two genes that encode two types of HMWgs known as x- and y-types (46). Since not all the *Glu-1* genes are transcriptionally active in bread or durum wheat, the numbers of HMW subunit proteins in bread wheat cultivars vary between 3 and 5 (49) and in durum wheat cultivars between 2 and 3 (50). On the other hand, the scenario for LMWgs and gliadins is much more complex. For example, based on sequence analysis, Huo et al. (51) identified 47 α-gliadin genes on the three common wheat subgenomes, of which 26 encode intact full-length proteins. Similarly, Qi et al. (52) reported 29 functional γ-gliadin genes in a single cultivar and Sabelli and Shewry (53) estimated 15–18 ω-gliadin genes in the common wheat cultivar “Chinese Spring” and five to ten genes in the tetraploid wheat cultivar “Langdon”. Through RNA sequencing, Wang et al. (54) identified 52 gliadin genes in the bread wheat cultivar “Xiaoyan 81” of which only 42 were active, and 25 were predicted to encode α-gliadins, 11 to encode γ-gliadins, one to encode δ-gliadin, and five to encode ω-gliadins. It is apparent from these earlier studies that immunogenic-gluten proteins (gliadins and LMWgs) are encoded by complex loci making it difficult to remove them *via* conventional approaches, such as induced mutagenesis or stacking of mutants *via* the genetic crossing of individual wheat plants containing gluten knockout mutants. Because, as mentioned, genes at these loci are arranged in tandem and are generally inherited as one locus; their elimination requires targeting the master control mechanism regulating gluten expression.

*Cis*- and *trans*-regulators of gluten genes were identified in the past [for a review, see (55)]. In particular, Sørensen demonstrated the role of epigenetic regulation (promoter methylation/de-methylation) in the transcriptional regulation of the prolamin genes in barley (56). Later work by the same researcher (57) and Radchuk et al. (58) validated these findings. Given this knowledge and the research in Arabidopsis on the role of DME in endosperm-specific demethylation of storage protein genes (59) and DRE2 in DME activation and its binding to DNA and demethylation (60), we tested the roles of DME and DRE2 in transcriptional derepression of wheat prolamins (21–23). The research of Zhu et al. (61) reconfirmed our findings and also demonstrated the importance of *cis-* and *trans*-regulation in wheat prolamin accumulation. The present research further validates the role of DME and DRE2 in the transcriptional regulation of prolamin genes in wheat endosperm. Further, the current research also reveals that these genes are vital for pollen development and viability as was observed in Arabidopsis (62, 63).

As emphasized earlier, the functions of specific genes should be directly validated in the crop of interest (64); hence we cloned *DME* and *DRE2* genes from the wheat genome, specifically from our cultivars of interest, “Chinese Spring” and “Kronos” (Figure 1, Supplementary Figure S2). Much like most of the genes in the wheat genome (64), the wheat *DME* and *DRE2* genes existed in triplicates with one copy on each subgenome (Figure 1). Since the homoeologous copies of a gene do not always exhibit functional redundancy or compensate for each other's loss (65, 66), we studied the expression patterns of these genes during early grain development (5, 7, 8, 10, and 13 DPA) and studied the effect of induced mutations in specific *DME* and *DRE2* homoeologues. This analysis improved our current understanding of the transcriptional interplay of *DME* and *DRE2* homoeologues and prolamin genes in the developing wheat endosperm.

Both *DME* and *DRE2* exhibited asymmetrical expression patterns among the homoeologous copies throughout the studied grain development period (6–30 DPA). The *DME* homoeologue on 5DL exhibited a dominant expression pattern throughout grain development in the meta-expression analysis and the expression analysis we performed *via* PCR-cloning and DNA sequencing (Figure 2). Similarly, *DRE2*'s 2AL-copy exhibited the highest expression at 6 and 14 DPA, and its 2DL-copy at 12 DPA (Figure 2). In the starchy endosperm, the expression patterns of *DME* and *DRE2* overlapped to some extent until 20 DPA (Figure 2). By contrast, they exhibited opposite expression patterns in the aleurone (Figure 2). Like *DME* and *DRE2*, the well-known wheat *reduced height-1* (*Rht-1*) gene provides an example of differential expression and function of homoeologous genes. The three homoeologous copies of this gene, *Rht-1A, Rht-1B*, and *Rht1-D*, that map to the wheat chromosomes 4A, 4B, and 4D, to some extent showed differential expression at the stem base and peduncles during the developmental process. Further, the mutations in these genes exhibit some overlapping and some unique functions (67). Another such example comes from a domestication gene *Q*. Its homoeologues, *5AQ, 5BQ*, and *5DQ*, map to chromosomes 5A, 5B, and 5D, respectively. The analysis of homeoalleles of the *Q* gene revealed that its three homoeologous copies had attained different fates in the polyploid wheat, such as *5AQ* hyperfunctionalized, *5Bq* pseudogenized, and *5Dq* subfunctionalized (68).

Our earlier research established DME and DRE2 as transitional regulators of prolamins in the wheat endosperm *via* gene silencing (21–23). In this study, to gain further insight into the roles of *DME* and *DRE2* homoeologues in the transcriptional regulation of prolamins, we compared the expression patterns of these genes with that of the expression patterns of immunogenic gluten proteins. Interestingly, we observed an opposite expression trend, which means the peaks of high gene expression for *DME* and *DRE2* opposed valleys of low gene expression for the immunogenic prolamins (Figure 2). However, this agrees with our hypothesis that *DME* and *DRE2* are expressed before the prolamin genes to induce their expression *via* promoter demethylation. Also, the transcript level for *DME* (a DNA glycosylase/lyase) is less than *DRE2* (a Fe/S cluster protein), and *DRE2*'s expression is less than the prolamin genes', which also made sense as the regulatory genes generally exhibit low expression compared to the structural genes (Figure 2) (69). The expression pattern of *DME* and *DRE2* genes was also compared to gluten protein accumulation levels at different grain developmental stages (Figure 3). Our analysis showed that the *DME*-5DL copy and the *DRE2*-2AL copy trigger the accumulation of the γ-gliadins and LMWgs at 8 DPA, at 10 DPA the *DME*-5AL and−5BL copies and the *DRE2*-2DL copy trigger accumulation α/β-gliadins, and at 13 DPA *DME*-5DL and *DRE2*-2AL and−2DL copies trigger the accumulation of ω-gliadins and HMWgs (Figure 3). The expression patterns of the prolamins observed in the present study coincided with earlier reports, such as Dupont et al. (70), who demonstrated that γ-gliadin transcripts accumulate as early as 6DPA and Pistón et al. (71), who showed that the γ-gliadins start to accumulate at 8DPA, α/β-gliadins at 10 DPA, and ω-gliadins at 12 DPA (Supplementary Figure S5). However, the transcriptional regulation of prolamins seems to differ significantly in barley where γ3-hordeins that correspond to wheat ω-5 gliadins accumulate in 2 mm developing grains and the rest of the hordeins D-hordeins (corresponding to wheat HMWgs), B1-hordeins (corresponding to wheat LMWgs), C-hordeins (corresponding to wheat γ-gliadins), and γ-hordeins (corresponding to wheat γ-gliadins) start to accumulate when the developing grains are 4 mm in size (Supplementary Figure S5). Interestingly *DME* exhibited a constitutive expression pattern in barley from 2 to 6 mm grains (Supplementary Figure S5).

In our analysis, we did not study the subgenome-specific expression of prolamin genes. However, in the past, 68% of the total gene expression of LMWgs was determined to occur from the “B” subgenome (72). Similarly, the genes of the “D” subgenome were shown to account for two-thirds of the total expression of the HMWgs, and for α-, ω-, and γ-gliadins, the gene copies of the “B” and “D” subgenomes were shown to dominate total gene family expression (72).

Further, mutants in specific *DME* and *DRE2* homoeologues tend to show reduced accumulation of specific gluten proteins, suggesting that different homoeologues are sub-functionalized to some extent (Figure 4, Supplementary Figure S2). For instance, plants with mutations in “A” subgenome *DME* homoeologue are more likely to lack ω-gliadins and “B” subgenome *DRE2* homoeologue are likely to be reduced in γ-gliadins (Figure 4, Supplementary Figure S2). It suggests *DME*-5A may be responsible for ω-gliadin gene promoter demethylation and *DRE2-2B* in promoting γ-gliadin promoter demethylation, setting the basis for specific targeting of protein groups and eliminating specific epitopes causing celiac disease and wheat allergy.

Interestingly, we observed that some of the mutations exhibited reduced transcript levels compared to the wild-type control (Supplementary Figure S3). These observations at first seemed unusual as we expected the transcript with an altered message due to substitutions or shorter transcript due to premature stop codon but not reduced content of the transcript. Among the five mutants that exhibited transcriptional suppression, three were in the “Kronos” background and two in the “Express” background (Supplementary Figure S3). The mutants in the “Kronos” background showed a higher level of suppression, which made sense as we studied the cumulative expression of *DME* homoeologues, and a more significant loss is expected in a tetraploid with one of the two copies of the gene mutated than in a hexaploid with one of the three copies of the gene silenced. Out of these three mutations, one was a nonsense mutation, the second was a splice site variant, and the third was a missense mutation (Supplementary Figure S3). The last two mutations exhibited more reduction in transcript level (Supplementary Figure S3). These observations conform to earlier findings where intercalary nonsense mutations (73) or substitutions (74) were shown to reduce transcript abundance.

Unlike the barley reduced-hordein *lys3a* mutant, later identified to encode barley Prolamin-box Binding Factor (BPBF) (34), *DME* mutants carrying premature stop codons or splice site variants, especially in the tetraploid wheat background, showed a reduction in anther size, locule size, number of fertile pollen grains, and pollen germination rate (Figure 4, Supplementary Figure S6). Zhu et al. have recently established the connection between promoter demethylation and different transcription factors in inducing prolamin gene expression and accumulation in grains (61). Congruently, in rice, Liu et al. (75) identified a *DME* family gene *ROS1* to determine the number of aleurone cell layers and rice's nutritional value *via* the transcriptional regulation of two putative aleurone differentiation-related transcription factors (75). Further, the reduction in pollen germination adversely affects the competitiveness of mutant pollen grains compared to wild-type pollen grains, which causes segregation distortion (62). Indeed, analysis of several hundred F_2_ plants from crosses between “A” and “B” subgenome *DME* mutants with premature stop codons yielded either single mutants or wild type alleles at both homoeologues (Supplementary Figure S4, Supplementary Table S5). Furthermore, a reduction in seedling vigor of the *DME* mutants with stop codons was also observed. Both of these observations indicate the vital role of *DME* in active DNA demethylation, which is crucial for seed viability, pollen germination, and reproduction in plants (76). These findings were consistent with observations in *Arabidopsis* (77) and rice (78). In particular, in rice, mutations in homologous gene ROS1 were defective in seed and pollen viability (78). Similarly, in *Arabidopsis*, Zhang et al. (77) demonstrated the importance of the DEM N-terminal domain for plant development and its conserved function in other dicots (77).

Interestingly, the mutation frequencies observed after screening about 3,500 M_2_s in the *DME* 5A and 5B copies in durum wheat “Kronos” were one mutation per 86 and 115 kb, respectively, and common wheat “Express” were one mutation per 86 and 53 kb, respectively (Table 1). Whereas, the mutation frequency in the common wheat *DME*-5D homoeologues was much lower, one mutation per 150 kb after screening more than 13,800 M_2_s (Table 1). These observed mutation frequencies were much lower than what was earlier reported by Slade et al. (27), i.e., one mutation per 24 kb in hexaploid wheat and one mutation per 40 kb in tetraploid wheat. This also indicates the vital roles of these genes—specifically, that of the *DME*-5B homoeologue in “Kronos” and *DME*-5D homoeologue in “Express.”

Many gluten proteins contribute to the unique textural and organoleptic properties of the wheat grain and derived products (79–81). Their lack or reduced amount in the generated wheat *DME* and *DRE2* mutants thus was expected to critically affect the end-use quality of derived products. Hard red spring wheat (“Express”) is used for leavened or unleavened bread preparation, and durum wheat (“Kronos”) is used to manufacture spaghetti and pasta. The present study did not suggest any major effects of eliminating specific gluten proteins on end-use quality based on the micro SRC analysis and SDS sedimentation test (Supplementary Table S4). Similarly, the study performed by Kieffer et al. demonstrated that it was possible to bake bread with good crumb and crust structures after eliminating all gluten except HMWgs from wheat flour. It was shown by baking bread from a mixture of the washed-out wheat flour residues (containing starch, soluble protein, fat, fibers, and minerals) and known quantities of recombinant HMWg subunits (HMWDx5 and HMWDy10). The dough kneaded from the mixture showed good elasticity and, after baking, resulted in bread rolls with reasonable volume and internal structure (20). Similar conclusions were reached when the flour derived from wheat transformants lacking one or more families of the gluten proteins baked into normal-looking bread loaves with characteristic organoleptic properties (17). In a similar study, removing the ω-gliadin, γ-gliadin, and LMWgs loci from the short arm of chromosome 1 of the D-subgenome (1DS) removed T-cell stimulatory epitopes from the proteome while maintaining technological properties (82). Our research on the silencing of wheat *DME* gene homoeologues, which affect the accumulation of gliadins and LMWgs, in a soft white winter wheat background, resulted in wheat lines with dough mixing properties comparable to hard red wheat genotypes (4). Precisely, these earlier studies suggested that deletions in genes encoding different gluten proteins in most cases bake into bread/cookies with reasonable textural and organoleptic properties. Therefore, to some extent, these experiments have answered the question about the utility of low-gluten and/or celiac-safe wheat genotypes in baking bread/cookies. Also, in a recent study, Guzmán-López et al. (83) showed that the bread derived from a low-gluten RNAi wheat line did not elicit the immune response in the DQ2.5-positive celiac patients, much like the gluten-free diet, under a short-term oral challenge. However, despite the fact that the reduced gluten mutants maintain reasonable end-use properties and exhibit reduced content of immunogenic proteins, inducing mutations in the *DME* and *DRE2* genes is an unviable approach due to the role of these genes in pollen viability and germination. Hence, the tissue-specific suppression of these genes or creating mutations in the promoters of these genes *via* gene editing could be an alternative solution.

## Data Availability Statement

The datasets presented in this study can be found in online repositories. The names of the repository/repositories and accession number(s) can be found below: https://www.ncbi.nlm.nih.gov/genbank/, JF683316-JF683318.

## Author Contributions

SRu, CM, and DW contributed to the conception and design of the study. NW, CM, CO, RB-A, JM, TA, SK, DW, and SRu performed the experiments. NW, CO, and SRu wrote the first draft of the manuscript. SRu, CR, SRe, and CM edited the manuscript. All authors contributed to the article and approved the submitted version.

## Funding

Financial support by NIH grant 2R42DK072721-02 and Life Sciences Discovery Fund grant 3143956-01, and Clemson Faculty Succeeds grant 1502211 is gratefully acknowledged.

## Conflict of Interest

The authors declare that the research was conducted in the absence of any commercial or financial relationships that could be construed as a potential conflict of interest.

## Publisher's Note

All claims expressed in this article are solely those of the authors and do not necessarily represent those of their affiliated organizations, or those of the publisher, the editors and the reviewers. Any product that may be evaluated in this article, or claim that may be made by its manufacturer, is not guaranteed or endorsed by the publisher.

## Supplementary Material

Supplementary Figure S1 Picture showing results of the microarray hybridization of the Chinese Spring BAC library filters with *DEMETER*-specific probe. Coordinates of positive BAC clones are shown on the filters.

Supplementary Figure S2 Diagrammatic representation of the wheat *DEMETER* gene to highlight the position of exons, specifically 4–10 (rectangular boxes), and show the location of *DEMETER* “B” homoeologue specific primer (4262) used for sequencing of PCR products **(A)**. Results of sequencing confirmed the zygosity of M_3_ plants obtained from heterozygous M_3_ seeds for the G/C to A/T mutation, changing Gly to Arg in the enzyme sequence **(B)**. Wt, wild type; Mu, mutant; Hz, heterozygote.

Supplementary Figure S3 Expression profiling of *DEMETER* (*DME*) using RNA extracted from the immature M_4_ grains of homozygous mutant plants using qRT-PCR (LightCycler^®^ 96). **(A)** The Ratio Bars chart shows the correspondence among ratios for each sample (mutant and wild type lines). *DME* mRNA level was normalized to *Actin*. Dotted circles indicate M_4_ plants with suppression of *DME* transcripts. **(B)** Table listing details [mutant ID, name, type, and crossing point (Cp)/threshold cycle (Ct)] of individual mutant lines and the wild type genotypes.

Supplementary Figure S4 **(A)** Diagrammatic representation of crosses made between the TILLING mutations identified in the “A” and “B” subgenome *DEMETER* homoeologues. Numbers in the grids represent total number of seeds obtained per cross. **(B)** The sequence of events after crossing of DEMETER single mutants obtained in “Express” and “Kronos” backgrounds for the “A” and “B” subgenome homoeologues. Na = data not available. I crossing = first crossing event and II crossing = second crossing event (duplication of the first event).

Supplementary Figure S5 Picture showing correspondence between *DEMETER* and the gluten gene expression and accumulation in the wheat grains. Expression pattern of the barely *DEMETER* and hordein genes in developing grains (2 to 6 mm size) studied *via* semi-quantitively PCR followed by densitometric analysis **(A)**. Transcript accumulation profiles for gliadin and glutenin subunit genes expressed in developing wheat endosperm (data derived from 67) **(B)**. Mass spectrometric analysis of gliadins and glutenins from developing wheat grains at 6, 8, 10, and 12 days post-anthesis [data derived from Pistón et al. (71)] **(C)**.

Supplementary Figure S6 Phenotypes of the selected TILLING mutants in the wheat *DEMETER* gene. These phenotypes are indicative of the background mutations.

Supplementary Table S1 List of homoeologues-specific *DEMETER* primers used for cytogenetic mapping and screening of TILLING libraries.

Supplementary Table S2 List of selected mutants checked for their genotypes and zygosities at the M_3_ stage. Immature and mature grains were harvested from the homozygous mutants to be checked for their transcriptional and protein profiles [modified from (22)].

Supplementary Table S3 List of wheat DEMETER mutants and their gluten profiles.

Supplementary Table S4 Results of the micro SRC analysis and other quality parameters of the selected wheat *DEMETER* mutants.

Supplementary Table S5 List of F_2_ plants selected for propagation in the greenhouse until maturity for further analysis [Modified from (22)].
