# Supplementary material for: Targeting Induced Local Lesions in the Wheat DEMETER and DRE2 Genes, Responsible for Transcriptional Derepression of Wheat Gluten Proteins in the Developing Endosperm
Source: Front Nutr. 2022 Mar 3;9:847635. doi: 10.3389/fnut.2022.847635 (PMC8928260; doi:10.3389/fnut.2022.847635)
Supplement: Supplementary Figure S1 — Picture showing results of the microarray hybridization of the Chinese Spring BAC library filters with DEMETER-specific probe. Coordinates of positive BAC clones are shown on the filters. [file Presentation_1.PPTX]

## Slide 1
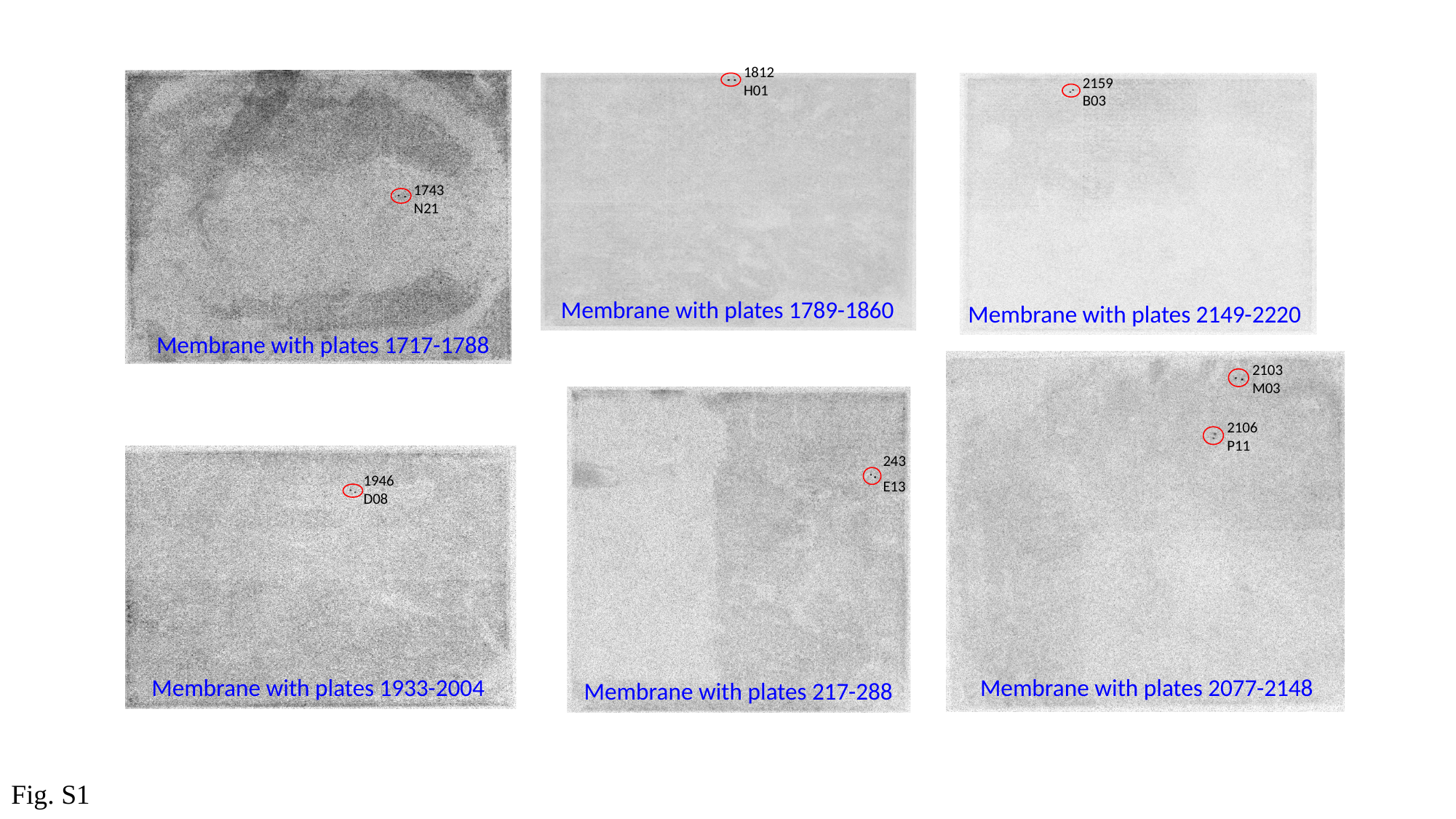

1812
H01
Membrane with plates 1789-1860
2159
B03
Membrane with plates 2149-2220
1743
N21
Membrane with plates 1717-1788
2103
M03
2106
P11
Membrane with plates 2077-2148
243
E13
Membrane with plates 217-288
1946
D08
Membrane with plates 1933-2004
Fig. S1

## Slide 2
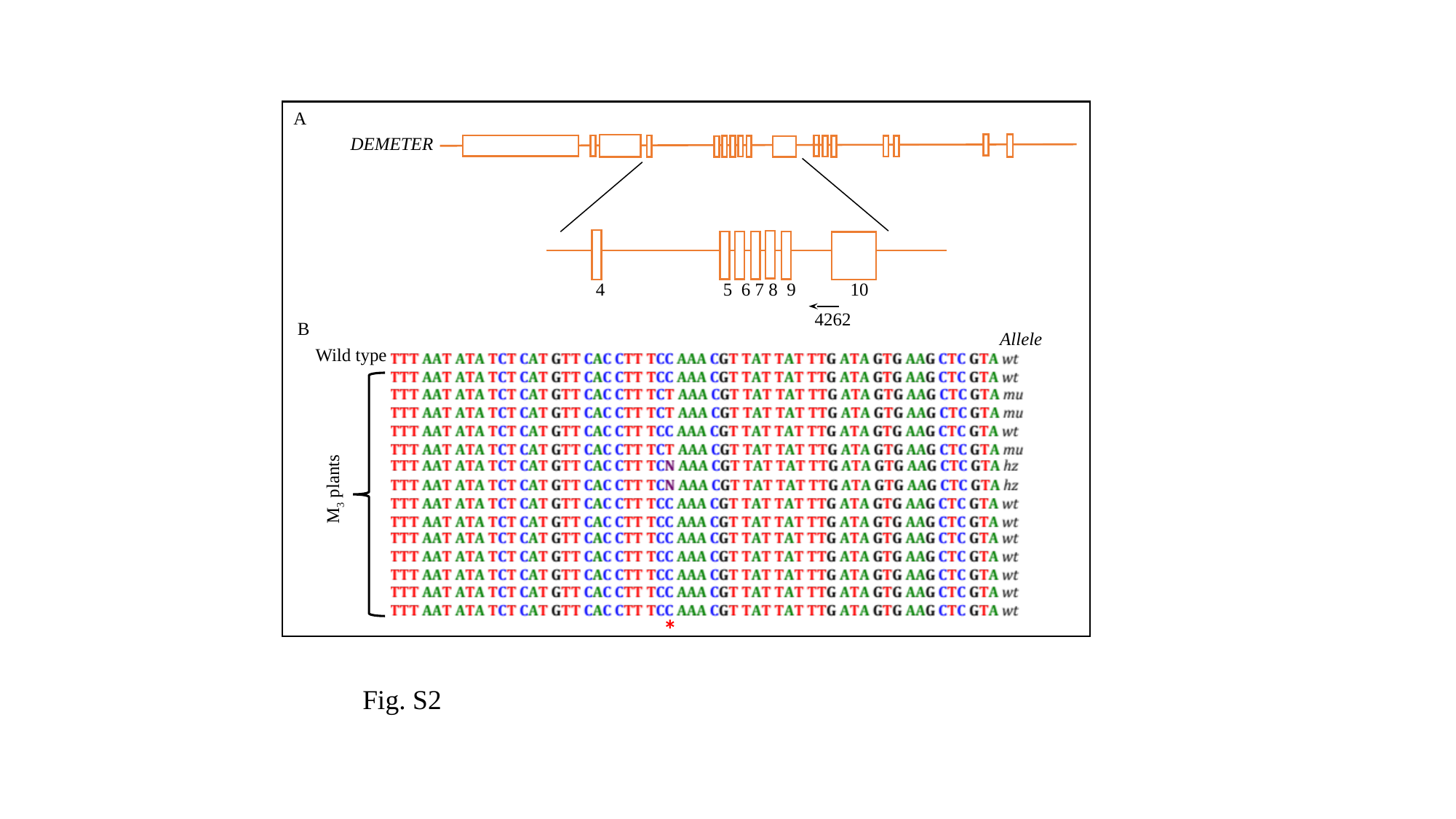

A
DEMETER
4 5 6 7 8 9 10
4262
B
Allele
Wild type
M3 plants
*
Fig. S2

## Slide 3
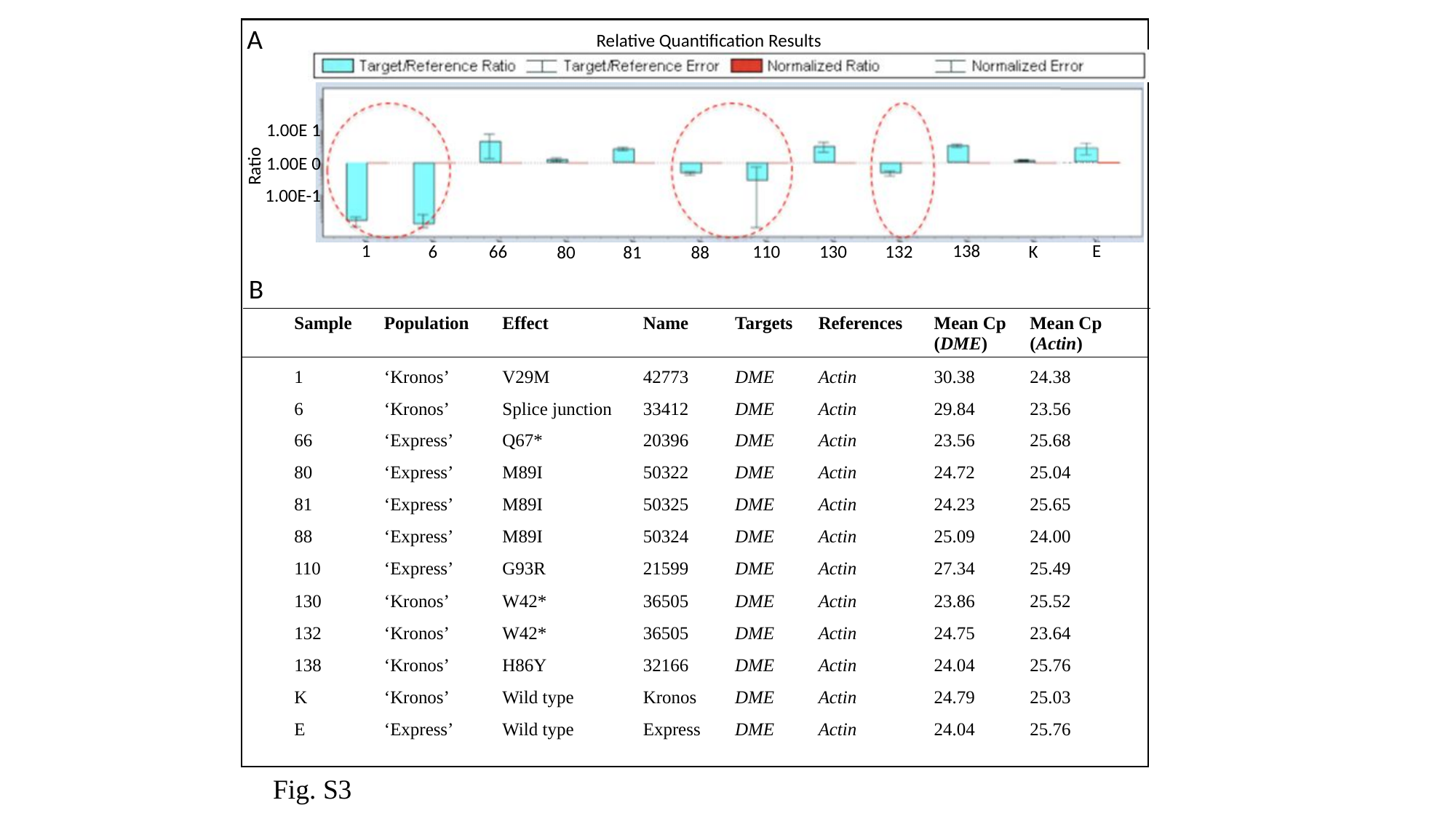

A
Relative Quantification Results
1.00E 1
1.00E 0
Ratio
1.00E-1
138
1
E
132
130
110
66
6
K
80
88
81
B
| Sample | Population | Effect | Name | Targets | References | Mean Cp (DME) | Mean Cp (Actin) |
| --- | --- | --- | --- | --- | --- | --- | --- |
| 1 | ‘Kronos’ | V29M | 42773 | DME | Actin | 30.38 | 24.38 |
| 6 | ‘Kronos’ | Splice junction | 33412 | DME | Actin | 29.84 | 23.56 |
| 66 | ‘Express’ | Q67\* | 20396 | DME | Actin | 23.56 | 25.68 |
| 80 | ‘Express’ | M89I | 50322 | DME | Actin | 24.72 | 25.04 |
| 81 | ‘Express’ | M89I | 50325 | DME | Actin | 24.23 | 25.65 |
| 88 | ‘Express’ | M89I | 50324 | DME | Actin | 25.09 | 24.00 |
| 110 | ‘Express’ | G93R | 21599 | DME | Actin | 27.34 | 25.49 |
| 130 | ‘Kronos’ | W42\* | 36505 | DME | Actin | 23.86 | 25.52 |
| 132 | ‘Kronos’ | W42\* | 36505 | DME | Actin | 24.75 | 23.64 |
| 138 | ‘Kronos’ | H86Y | 32166 | DME | Actin | 24.04 | 25.76 |
| K | ‘Kronos’ | Wild type | Kronos | DME | Actin | 24.79 | 25.03 |
| E | ‘Express’ | Wild type | Express | DME | Actin | 24.04 | 25.76 |
Fig. S3

## Slide 4
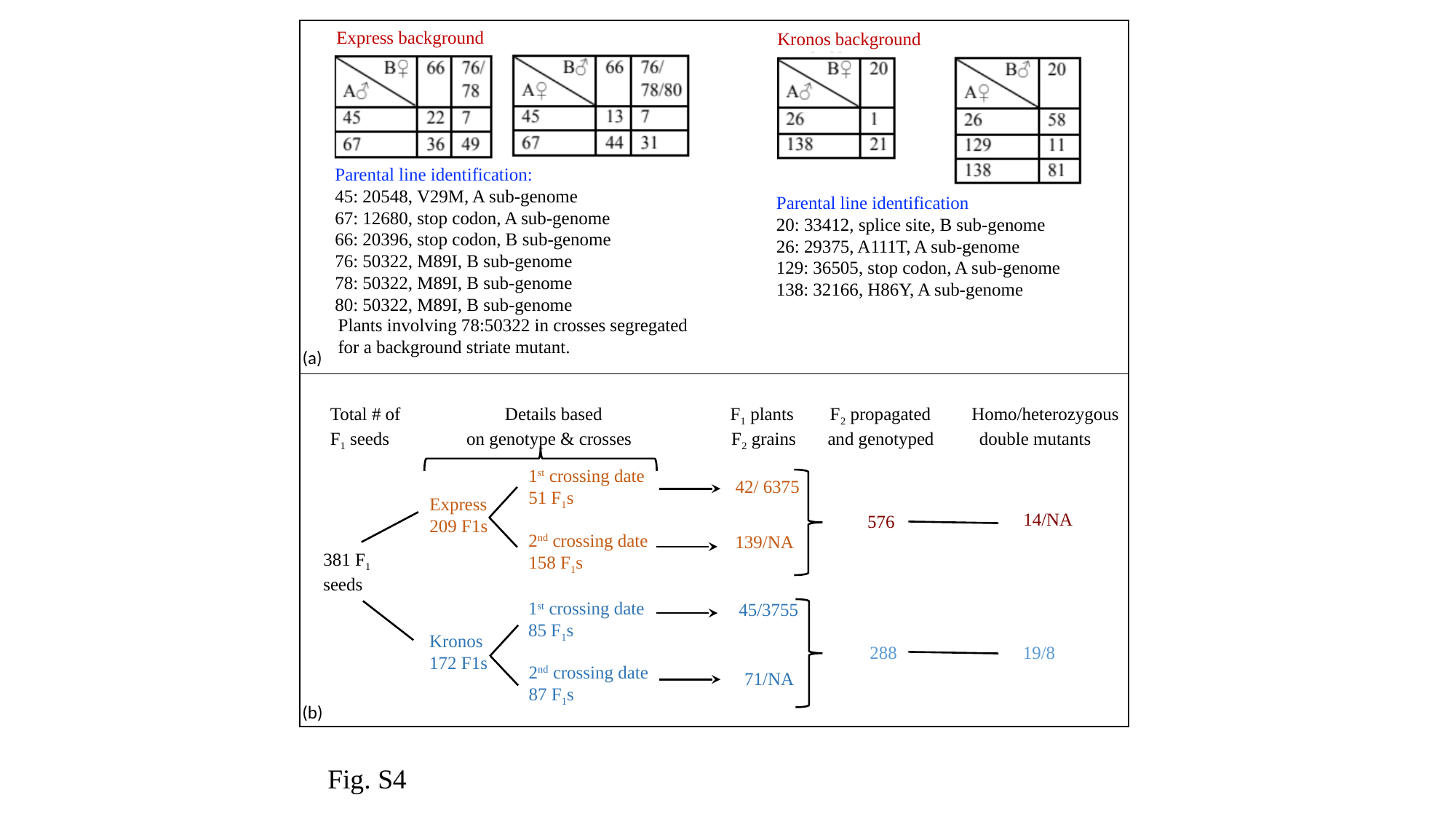

Express background
Kronos background
Parental line identification:
45: 20548, V29M, A sub-genome
67: 12680, stop codon, A sub-genome
66: 20396, stop codon, B sub-genome
76: 50322, M89I, B sub-genome
78: 50322, M89I, B sub-genome
80: 50322, M89I, B sub-genome
Parental line identification
20: 33412, splice site, B sub-genome
26: 29375, A111T, A sub-genome
129: 36505, stop codon, A sub-genome
138: 32166, H86Y, A sub-genome
Plants involving 78:50322 in crosses segregated
for a background striate mutant.
(a)
 Total # of Details based 	 F1 plants F2 propagated Homo/heterozygous
 F1 seeds 	 on genotype & crosses F2 grains and genotyped double mutants
1st crossing date
51 F1s
42/ 6375
14/NA
576
139/NA
 45/3755
19/8
288
71/NA
Express
209 F1s
2nd crossing date
158 F1s
381 F1
seeds
1st crossing date
85 F1s
Kronos
172 F1s
2nd crossing date
87 F1s
(b)
Fig. S4

## Slide 5
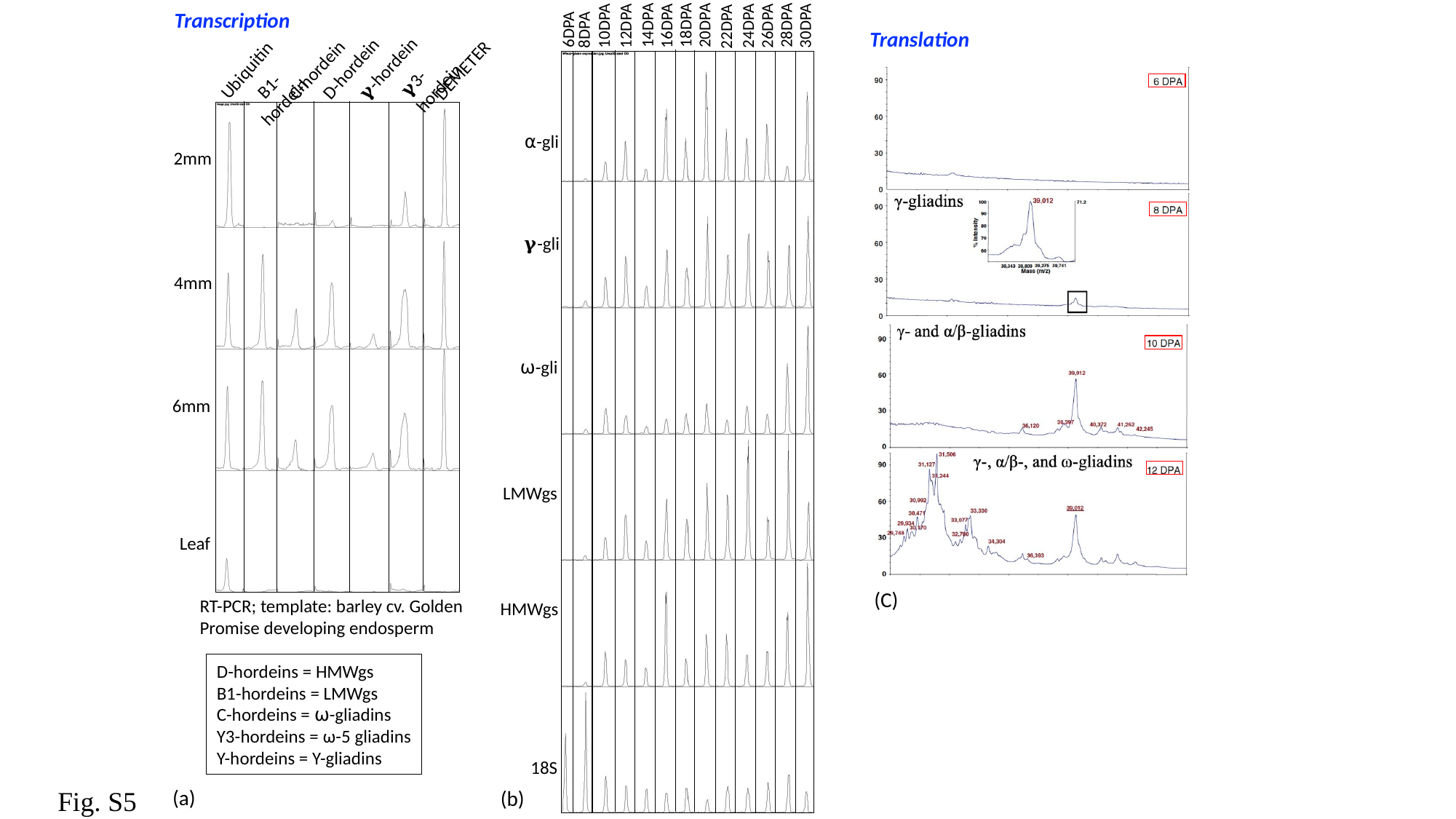

Transcription
18DPA
14DPA
20DPA
28DPA
10DPA
16DPA
24DPA
30DPA
12DPA
26DPA
22DPA
6DPA
8DPA
Translation
𝛄3-hordein
𝛄-hordein
D-hordein
C-hordein
Ubiquitin
DEMETER
 B1-hordein
⍺-gli
2mm
𝛄-gli
4mm
⍵-gli
6mm
LMWgs
Leaf
(C)
RT-PCR; template: barley cv. Golden Promise developing endosperm
HMWgs
D-hordeins = HMWgs
B1-hordeins = LMWgs
C-hordeins = ⍵-gliadins
Y3-hordeins = ω-5 gliadins
Y-hordeins = Y-gliadins
18S
Fig. S5
(a)
(b)

## Slide 6
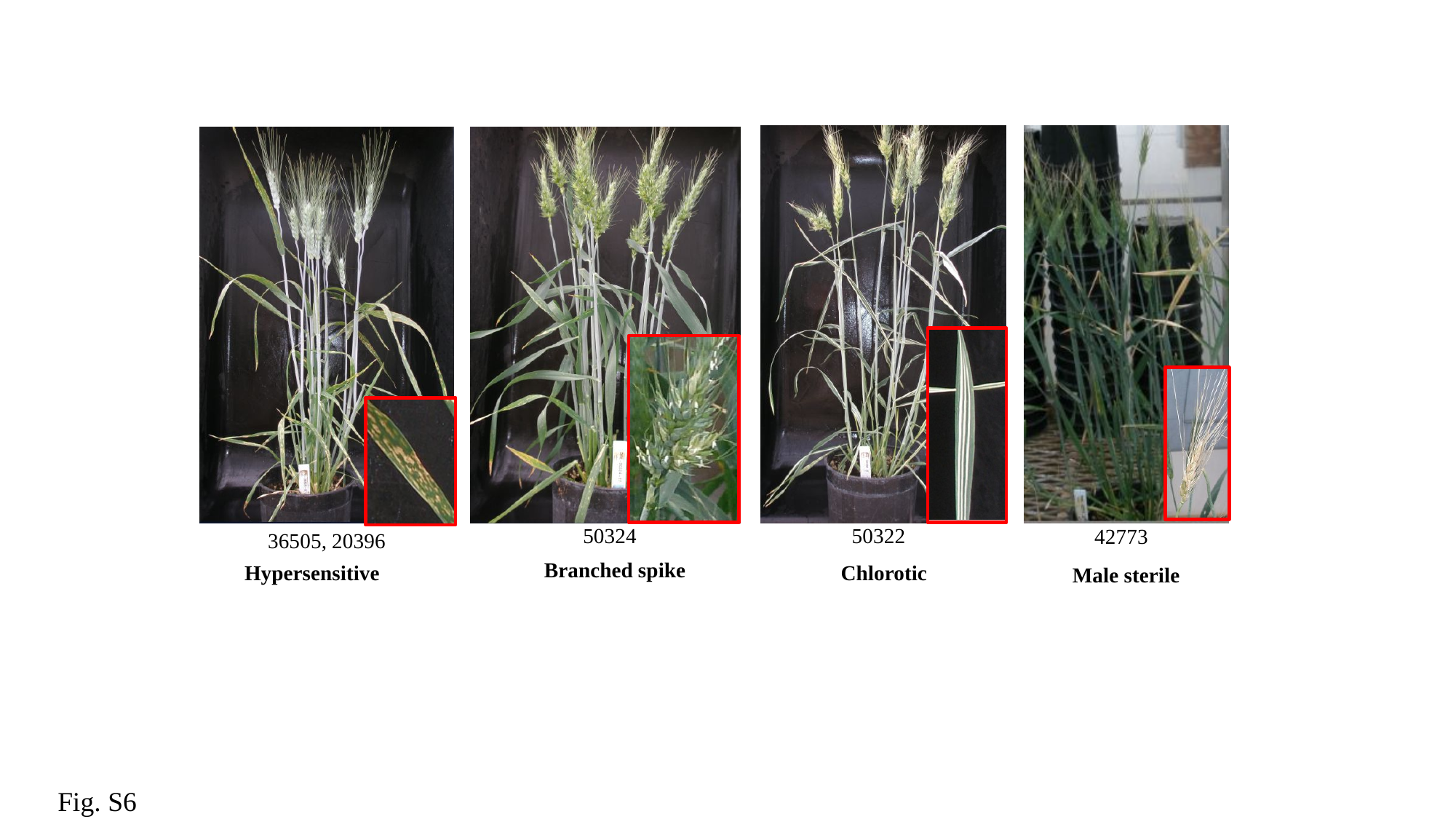

50324
50322
42773
36505, 20396
Branched spike
Hypersensitive
Chlorotic
Male sterile
Fig. S6
