# Supplementary material for: Targeting Induced Local Lesions in the Wheat DEMETER and DRE2 Genes, Responsible for Transcriptional Derepression of Wheat Gluten Proteins in the Developing Endosperm
Source: Front Nutr. 2022 Mar 3;9:847635. doi: 10.3389/fnut.2022.847635 (PMC8928260; doi:10.3389/fnut.2022.847635)
Supplement: Supplementary Table S1 — List of homoeologues-specific DEMETER primers used for cytogenetic mapping and screening of TILLING libraries. [file Table_1.DOCX]

**Table S1**: List of homoeologues-specific *DEMETER* primers used for cytogenetic mapping and screening of TILLING libraries.

| **Primer designation** | **Primer sequence (5'-3')** | **Product size (bp)** | **Primer allocation** |
| --- | --- | --- | --- |
| **Primers for cytogenetic mapping of wheat *DEMETER* homoeologues** | | | |
| 5A_2159_1:F | CACAATTAGTTGAGACGGGAAT | 200 | Intron13 |
| 5A_2159_1:R | CCTTCAAGCCTGATTGATGC |  |  |
| 5A_2159_2:F | ACCTGAAGTTCCTGCTGACA | 303 | Exon3-Intron3 |
| 5A_2159_2:R | TGCCCACATGTCCATATGACTA |  |  |
| 5B_1946_1:F | TCAAAAAGCAAATTCTGAACTCC | 370 | Exon3-Intron3 |
| 5B_1946_1:R | TTGCCCACATGTCTAAATACAGAAC |  |  |
| 5B_1946_2:F | ACAGAAATACCTCTGGCCTCGATTATGC | 546 | Exon7-Intron9 |
| 5B_1946_1:R | AAATATCATCAGTCGCTGCCGTCAAG |  |  |
| 5D_2106_1:F | ACAAAACTAGTGGGAACAGCAG | 528 | Introns13-15 |
| 5D_2106_1:R | CGTGAATTAATCCATGGAGTAGAT |  |  |
| 5D_2106_2:F | TCAAGACCCTATGAGTCCATAAC | 370 | Intron4 |
| 5D_2106_2:R | AGACCTATCACAAGAAAACTAATGG |  |  |
| **Primers for the screening of Kronos and Express TILLING libraries** | | | |
| DME_A_4259F | GGGAAGTTTGCATGGTTGACTGAAATAA | 1050 (K; E) | Intron 4-9 |
| DME_A_4261R | GGGAAATATTATCAGTCGATGCCATCAAA |  |  |
| DME_B_4260F | TAAAAGGGTTATTCTAAAAGTTATATTATGCG | 1045 (K) | Intron 4-9 |
| DME_B_3910F | TGTGTGCGTCTTTTGACACTCCAT | 855 (E) | Exon 5-Intron 9 |
| DME_B_4262R | AAATATCATCAGTCGCTGCCGTCAAG |  |  |
| DME_D_1555F | CCTTAGATGAATGTTTTTGGCGAAC | 1008 | Intron 4-9 |
| DME_D_2514R | ATACACAGTTCCACAGGAAACTCGA |  |  |
