# Supplementary material for: Targeting Induced Local Lesions in the Wheat DEMETER and DRE2 Genes, Responsible for Transcriptional Derepression of Wheat Gluten Proteins in the Developing Endosperm
Source: Front Nutr. 2022 Mar 3;9:847635. doi: 10.3389/fnut.2022.847635 (PMC8928260; doi:10.3389/fnut.2022.847635)
Supplement: Supplementary Table S2 — List of selected mutants checked for their genotypes and zygosities at the M3 stage. Immature and mature grains were harvested from the homozygous mutants to be checked for their transcriptional and protein profiles [modified from (22)]. [file Table_2.DOCX]

**Table S2.** List of selected mutants checked for their genotypes and zygosities at the M_3_ stage. Immature and mature grains were harvested from the homozygous mutants to be checked for their transcriptional and protein profiles (modified from Rustgi et al., 2014).

| ID | | Mutation | Effect | Zygosity determined at M_2_ | # of plants checked | Zygosity determined at M_3_ | Protein | qRT-PCR |
| --- | --- | --- | --- | --- | --- | --- | --- | --- |
| DME_5A_Kro | **36505^b^** | G354A | W42* | Het | 10 | Mutant, wildtype and heterozygote |  | supression |
|  | **32166** | C642T | H86Y | Het | 20 | Mutant and wildtype | gamma gli |  |
|  | 17186 | G663A | G93R | Het | 20 | Mutant, wildtype and heterozygote |  |  |
|  | 33322 | G669A | splice junction | Het | 10 | Wildtype and heterozygote |  |  |
|  | **29375** | G797A | A111T | ? | 20 | Mutant, wildtype and heterozygote |  |  |
| DME_5B_Kro | 42773^d^ | G308A | V29M | Hom | 10 | Mutant |  | supression |
|  | **33412** | G667A | splice junction | Het | 20 | Mutant, wildtype and heterozygote | gamma & alpha gli | supression |
|  | 36182 | C787T | P108L | Hom | 5 | Wildtype |  |  |
| DME_5A_Exp | **20548** | G313A | V29M | Hom | 5 | Mutant |  |  |
|  | **12680** | G354A | W42* | Het | 20 | Mutant, wildtype and heterozygote |  |  |
|  | 15945 | G480A | splice junction | Het | 20 | Mutant, wildtype and heterozygote |  |  |
|  | 15246 | G653A | M89I | Het | 20 | Mutant, wildtype and heterozygote |  |  |
|  | 22471 | C658T | T91M | Het | 20 | Mutant and wildtype |  |  |
|  | 14773 | G664A | G93E | Het | 20 | Mutant and wildtype |  |  |
|  | 46066 | G669A | splice junction | Hom | 5 | Wildtype |  |  |
|  | 46332 | G795A | R110K | Het | 20 | Wildtype |  |  |
|  | 46354 | G669A | splice junction | Hom | 4 | Mutant |  |  |
| DME_5B_Exp | 22914 | G307A | splice junction | Het | 20 | Mutant, wildtype and heterozygote |  |  |
|  | 20397 | C500T | Q67* | Hom | 5 | Mutant | omega gli |  |
|  | **20396^b^** | C500T | Q67* | Het | 20 | Mutant, wildtype and heterozygote | omega gli |  |
|  | **50322^a^** | G651A | M89I | Hom | 5 | Mutant |  |  |
|  | 50325 | G651A | M89I | Hom | 5 | Mutant |  |  |
|  | 50324^c^ | G651A | M89I | Het | 20 | Mutant, wildtype and heterozygote |  | supression |
|  | 21599 | G661A | G93R | Het | 20 | Mutant, wildtype and heterozygote |  | supression |
| DME_5D_Exp | 13105 | C221T | L9F | Hom | 2 | Mutant |  |  |
|  | 49004 | C278T | P28S | Hom | 2 | Mutant |  |  |
|  | 19793 | C273T | A26V | Hom | 2 | Mutant |  |  |
|  | 22244 | G363A | D30N | Het | 8 | Wildtype |  |  |
|  | 50449 | G363A | D30N | Het | 6 | Mutant, wildtype and heterozygote |  |  |
|  | 50503 | G197A | E1K | Het | 6 | Mutant, wildtype and heterozygote |  |  |
|  | 51464 | C367T | T31I | Het | 6 | Mutant, wildtype and heterozygote |  |  |
|  | 49066 | C367T | T31I | Het | 6 | Mutant, wildtype and heterozygote |  |  |
|  | 15603 | C203T | L3F | Het | 6 | Mutant, wildtype and heterozygote |  |  |

Red font = stocks showing incorrect genotypes in M_3_ generation (not corresponding with the genotypes determined in M_2_)

Blue font = stocks selected for crossing

a = striata mutation in background

b = necrotic mutation in background

c = mutation causing supernumerary spikeles in background

d = mutation causing male sterility in background

Rustgi, S., Wen, N., Osorio, C., Brew-Appiah, R. A., Wen, S., Gemini, R., et al. (2014). Natural dietary therapies for the ‘gluten syndrome’. Scientia Danica, Series B, Biologica 3:1–87
