# Supplementary material for: Targeting Induced Local Lesions in the Wheat DEMETER and DRE2 Genes, Responsible for Transcriptional Derepression of Wheat Gluten Proteins in the Developing Endosperm
Source: Front Nutr. 2022 Mar 3;9:847635. doi: 10.3389/fnut.2022.847635 (PMC8928260; doi:10.3389/fnut.2022.847635)
Supplement: Supplementary Table S5 — List of F2 plants selected for propagation in the greenhouse until maturity for further analysis [Modified from (22)]. [file Table_5.DOCX]

**Table S5**. List of F_2_ plants selected for propagation in the greenhouse until maturity for further analysis (Modified from Rustgi et al., 2014).

| **Express** | **DME_5B** | **DME_5A** |  |
| --- | --- | --- | --- |
| Plant # | 50322, M89I, G651A [D3] | 20548, V29M, G313A [D2] | Class |
| 72 | MM | MW | aaBb |
| 73 | MM | MM | aabb |
| 80 | MM | MM | aabb |
| 85 | MM | MW | aaBb |
| 86 | MM | MW | aaBb |
| 96 | MM | MM | aabb |
| 75 | MM | MW | aaBb |
| 76 | MW | MW | AaBb |
| 83 | MW | MM | Aabb |
| 87 | MW | MW | AaBb |
| 88 | MW | MM | Aabb |
| 90 | MW | MW | AaBb |
| 91 | MW | MW | AaBb |
| 94 | MW | MW | AaBb |
| **Kronos** | **DME_5B** | **DME_5A** |  |
| Plant # | 33412, splice site, G667A [D3] | 29375, A111T, G797A [D3] | Class |
| 145 | MM | MM | aabb |
| 148 | MM | MM | aabb |
| 175 | MW | MM | Aabb |
| 176 | MW | MM | Aabb |
| 196 | MW | MM | Aabb |
| 204 | MW | MM | Aabb |
| 205 | MW | MM | Aabb |
| 209* | MW | MM | Aabb |
| 210 | MW | MM | Aabb |
| 213 | MM | MM | aabb |
| 218 | MW | MM | Aabb |
| 227 | MM | MW | aaBb |
| 228 | MM | MM | aabb |
| 238 | MM | MM | aabb |
| 241 | MW | MM | Aabb |
| 244 | MM | MW | aaBb |
| 251 | MW | MW | AaBb |
| 253 | MM | MM | aabb |
| 254 | MW | MW | AaBb |
| 256 | MM | MM | aabb |

*Plant died at seedling stage

D2 = HhH-GPD domain

D3 = 4Fe-4S binding domain

M = mutant allele; W = wild type allele

Rustgi, S., Wen, N., Osorio, C., Brew-Appiah, R. A., Wen, S., Gemini, R., et al. (2014). Natural dietary therapies for the ‘gluten syndrome’. Scientia Danica, Series B, Biologica 3:1–87
